# Human antibody targeting Crimean-Congo hemorrhagic fever virus glycoprotein 38 protects mice against heterologous virus challenge

**DOI:** 10.1172/JCI191440

**Published:** 2026-03-31

**Authors:** Nathaniel S. Chapman, Viktoriya Borisevich, Nurgun Kose, Luke Myers, Stephen Priest, Éric Bergeron, Elena Trigo Esteban, María Paz Sánchez-Seco, José Melero, Thomas W. Geisbert, Robert W. Cross, James E. Crowe

**Affiliations:** 1 Department of Pathology, Microbiology and Immunology and; 2 The Vanderbilt Center for Antibody Therapeutics, Vanderbilt University Medical Center, Nashville, Tennessee, USA.; 3 Galveston National Laboratory and; 4 Department of Microbiology and Immunology, The University of Texas Medical Branch, Galveston, Texas, USA.; 5 Department of Pharmaceutical and Biomedical Sciences, College of Pharmacy, University of Georgia, Athens, Georgia, USA.; 6 Viral Special Pathogens Branch, Division of High-Consequence Pathogens and Pathology, Centers for Disease Control and Prevention, Atlanta, Georgia, USA.; 7 National Referral Centre for Tropical Diseases and International Health, Hospital La Paz–Carlos III, IdIPaz, CIBERInfec, Madrid, Spain.; 8 Laboratory of Arbovirus and Imported Viral Diseases, National Center of Microbiology, and; 9 Centro Nacional de Microbiología, Instituto de Salud Carlos III, Madrid, Spain.; 10 Department of Pediatrics, Vanderbilt University Medical Center, Nashville, Tennessee, USA.

**Keywords:** Infectious disease, Virology, Adaptive immunity, Antigen, Immunoglobulins

## Abstract

Crimean-Congo hemorrhagic fever virus (CCHFV) is an emerging arboviral and zoonotic bunyavirus. CCHFV can infect livestock, wild animals, and humans. Here, we report the isolation of a panel of mAbs from the B cells of an immune individual following a natural nosocomial infection. We determined that the panel comprised antibodies that bound to 2 glycoproteins: (a) the carboxy-terminal glycoprotein (Gc) that serves as the fusion protein and (b) the glycoprotein 38 (GP38). By antibody variable gene analysis, we identified genetic diversity in the B cell response to CCHFV within a single donor for both Gc- and GP38-specific responses. Protection against most bunyavirus-associated diseases is mediated principally by neutralizing antibodies, but here, we found that neutralization activity was not associated with protection. Gc-specific antibodies to diverse antigenic sites neutralized only weakly and did not protect against heterologous virus challenge. GP38-specific antibodies bound to 2 dominant antigenic sites on the glycoprotein. Although GP38-specific antibodies did not neutralize the virus, one mediated protection against heterologous virus challenge in an experimental model of infection in mice primarily by complement-mediated activity. These studies support the development of protective CCHFV countermeasures against GP38.

## Abstract 2

A human monoclonal antibody directed to the GP38 protein of Crimean-Congo hemorrhagic fever virus (CCHFV) does not neutralize the virus but mediates protection against heterologous virus challenge in an experimental model of infection in mice. The studies reveal novel aspects of mechanisms of bunyavirus immunity.

## Introduction

Crimean-Congo hemorrhagic fever virus (CCHFV) is an emerging arbovirus and zoonotic threat to human health with epidemic potential (1–3). CCHFV was first identified in the Crimean region in 1944 and is now present in diverse geographical regions (1). CCHFV has been detected in the Balkans, Turkey, in other countries around the Mediterranean Sea, in the northwestern region of China, central Asia, Africa, the Middle East, and India (1, 2). The virus is typically transmitted by hard ticks (Ixodidae; most commonly, *Hyalomma spp.*) (1, 2). Many domesticated animals, including goats, sheep, and cattle, are permissive hosts (3). While human-to-human transmission has been documented, humans are more often infected by contact with ticks or animal blood or animal by-products (2–4). When infected, humans can present with sudden onset symptoms including headache, high fever, stomach pain, and vomiting (2–4). Disease progression may include progressive flushing of the face, neck, and chest and conjunctivitis. In more severe cases, extensive coalescing hemorrhagic manifestations in the skin and bleeding defects can occur (3). Mortality rates range from 3% to 30% (3). Concern over epidemic outbreaks has increased with the onset of climate change leading to expanded vector prevalence (3). As the habitable zones of the vectors increase, the virus has been detected in new regions, including as far north as Spain, France, and Portugal (3, 5–7). This trend of geographic spread may continue if the climate continues to warm. Given the high mortality rate, spread to new regions, and potential to be leveraged as a bioterrorism agent, WHO has designated CCHFV as a priority pathogen for urgent research and therapeutic development (8).

CCHFV, a member of the Bunyaviricetes class, Hareavirales order, *Nairoviridae* family*,* and *Orthonairovirus* genus (9, 10), has a tripartite RNA genome containing large, medium (M), and small gene segments (3, 10). The M segment encodes for the following viral envelope glycoproteins: Gn (the amino-terminal glycoprotein), Gc (the carboxy-terminal glycoprotein), GP38 (a glycosylated viral protein with a molecular weight of ~38 kDa), and a mucin-like domain (MLD) (11, 12). The genomes of CCHFV strains are diverse. For example, regarding the M segment, the amino acid sequences of 13 different strains isolated in diverse geographic and temporal settings differ by 27% (11).

Gc and Gn are the 2 glycoproteins on the viral surface and are formed from a precursor molecule that is cleaved by host proteases (12, 13). The Gc/Gn glycoproteins allow for viral attachment, entry, fusion at low pH, and assembly (10, 14–16). CCHFV Gc is a class II fusion protein that trimerizes to initiate fusion between host and viral membranes (17). Little is known regarding the function of CCHFV Gn and the accessory proteins encoded by the M segment, but it has been speculated that Gn may act as a cap to prevent premature exposure of the Gc protein, as in related viruses (18). A third protein, GP38, is derived from the pre-Gn precursor. GP38 has been observed in a secreted form and on the cell and viral surface (19). GP38 also plays a role in Gc trafficking and packaging the virus (16). More recently, however, GP38 was suggested to have toxin-like activity by inducing endothelial barrier dysfunction and vascular leak (20).

It has proven difficult for investigators to isolate potently neutralizing human mAbs to bunyavirus Gc glycoprotein. In some cases, such as for Rift Valley fever virus (RVFV), Gc-specific antibodies mediate only partial in vivo protection at best (21–23). Similar phenotypes have been noted in studies of a panel of Gc-specific murine mAbs (19, 24, 25). Data from murine and nonhuman primate experimental vaccine trials show that high serum neutralizing antibody titers do not associate with protection following immunization with protein (Gc and Gn) subunit or DNA-based candidate vaccines, respectively (26, 27). In contrast, animals that possess high levels of nonneutralizing serum antibodies recognizing GP38 or nucleocapsid protein (NP) exhibit protection (28–31).

Here, we characterized the B cell response to CCHFV infection in an individual who was infected in a healthcare setting to investigate the diversity of antibody genes induced by CCHFV infection and the mechanisms of antibody-mediated protection. We found most of the memory B cells induced by infection in this individual bound to Gc or GP38. We did not identify any human mAbs to Gn. We also did not identify any antibodies that bind to such complex quaternary epitopes, despite using Gc/Gn/GP38 antigens expressed in cells. Most of the CCHFV Gc-specific antibodies we isolated neutralized virus only weakly and provided no protection against heterologous challenge. We focused on a GP38-specific antibody (designated CCHF-82) that provided protection when administered as pre- or postexposure prophylaxis in a lethal mouse model of infection. These results agree with those of previous studies (19, 32–35). The protective human GP38-specific mAb CCHF-82 competed for binding to GP38 with a previously isolated murine mAb, 13G8, suggesting that it recognizes a common protective major antigenic site. Moreover, we provide evidence that the protective capacity of CCHF-82 is driven principally by complement-mediated activity. We observed diverse genetic antibody heavy- and light-chain gene usage in the CCHFV-reactive antibody repertoire in an individual soon after recovery from disease. The findings suggest CCHFV infection induced both protective nonneutralizing and nonprotective neutralizing antibodies to heterologous challenge in the repertoire of this convalescent individual.

## Results

### Human antibodies to diverse strains of CCHFV.

**Figure 1:**
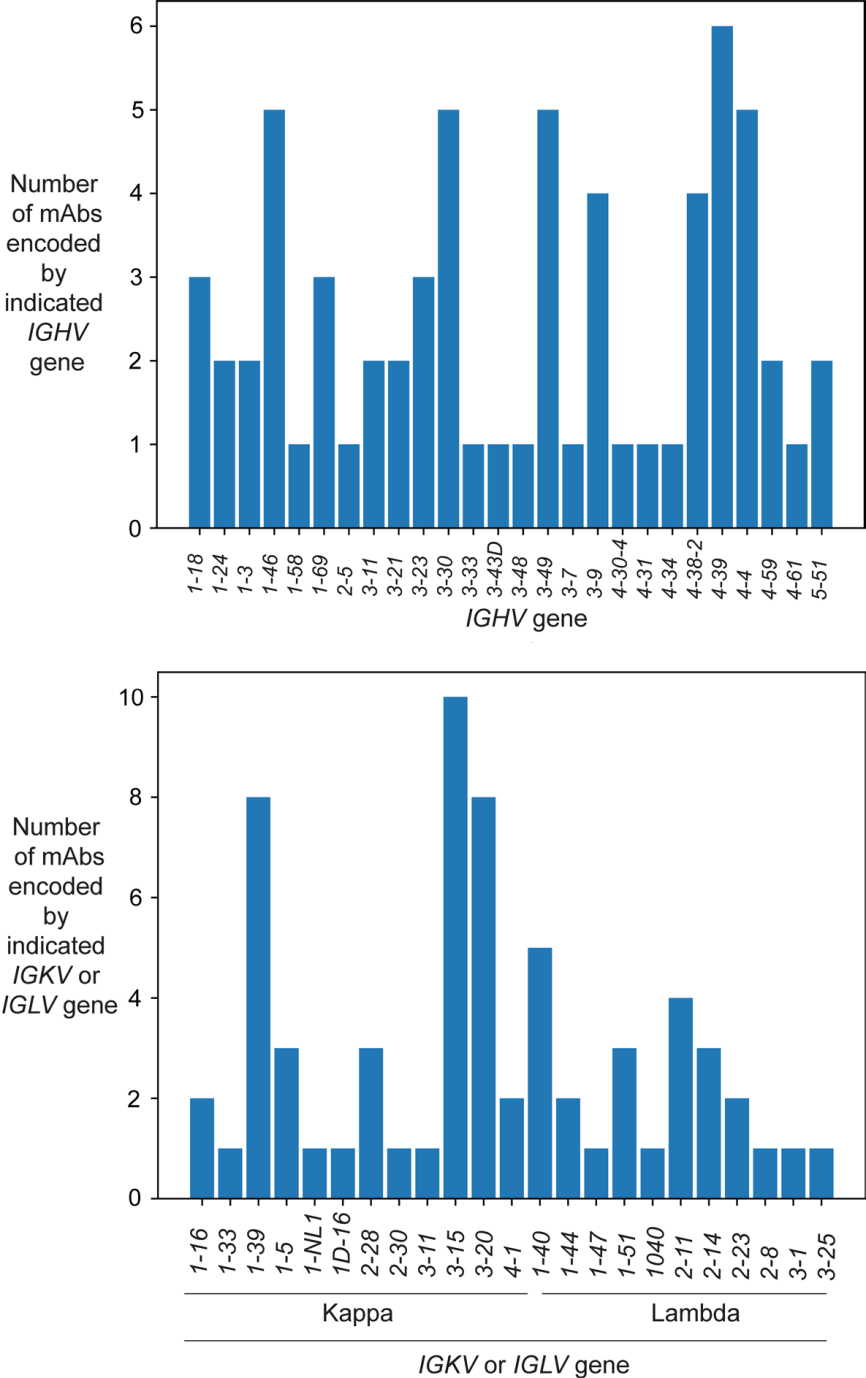
Diverse antibody V gene usages from a human survivor of CCHFV. A panel of 66 mAbs was isolated from a survivor of CCHFV using the hybridoma method. Histogram plots showing the relative abundance of human germline heavy- and light-chain V genes inferred from IMGT (the international ImMunoGeneTics information system) analysis of antibodies within our panel from 1 human survivor.

We isolated a panel of 66 human antibodies against CCHF from the PBMCs of a survivor (5) using the hybridoma method from 2 EBV transformation campaigns. In the first campaign, we isolated mAbs sequentially named CCHF-2 through CCHF-137, which form the basis of most experimentation in this report. The mAbs CCHF-144 through CCHF-275 were isolated in the second transformation campaign with another vial of PBMCs from the same donor. These antibodies from the second part of the panel were studied in select experiments. The individual had been infected with a strain of CCHF closely related to strain ArD39554 (5). Thus, of the laboratory isolates, IbAr10200 is the most closely related in sequence. The primary screen to identify CCHFV-reactive clones used in this effort was the following: an M segment–encoding plasmid with an insert based on the sequence of the CCHFV IbAr10200 strain was transiently transfected into human cells derived from the HEK293 cell line (Expi293F) for 48 hours so that GP38, Gc, and Gn were all expressed. Supernatants were collected from cultured B cells transformed with EBV, hybridoma cell lines created by the fusion of transformed B cells with a myeloma cell line, and hybridoma clones isolated by single-cell sorting from hybridoma lines. Supernatants were screened using a high-throughput flow cytometer (iQue) for the presence of antibodies binding to CCHFV antigens expressed from the transfected M segment plasmid DNA in cells. We also screened the supernatant for antibodies binding to a recombinant form of soluble Gn protein with a C-terminal 6xHis tag expressed in mammalian HEK293 cells (corresponding to M segment amino acids 520–690 of the full-length glycoprotein precursor [GPC] of the M segment of CCHFV; The Native Antigen Company). However, we did not identify any B cell lines that secreted antibodies that bound Gn (data not shown). We determined the antibody variable gene sequences encoding the antibodies isolated from memory B cells of this convalescent individual and grouped the mAbs based on heavy- and light-chain variable V gene usage (Figure 1 and Supplemental Table 1; supplemental material available online with this article; https://doi.org/10.1172/JCI191440DS1). We observed many V genes of human heavy and light chains participating in the response to CCHFV infection. Furthermore, limited genetic overlap was observed in the variable genes used in the panel, suggesting that we did not fully interrogate the CCHFV-reactive repertoire even within this convalescent individual.

**Figure 2:**
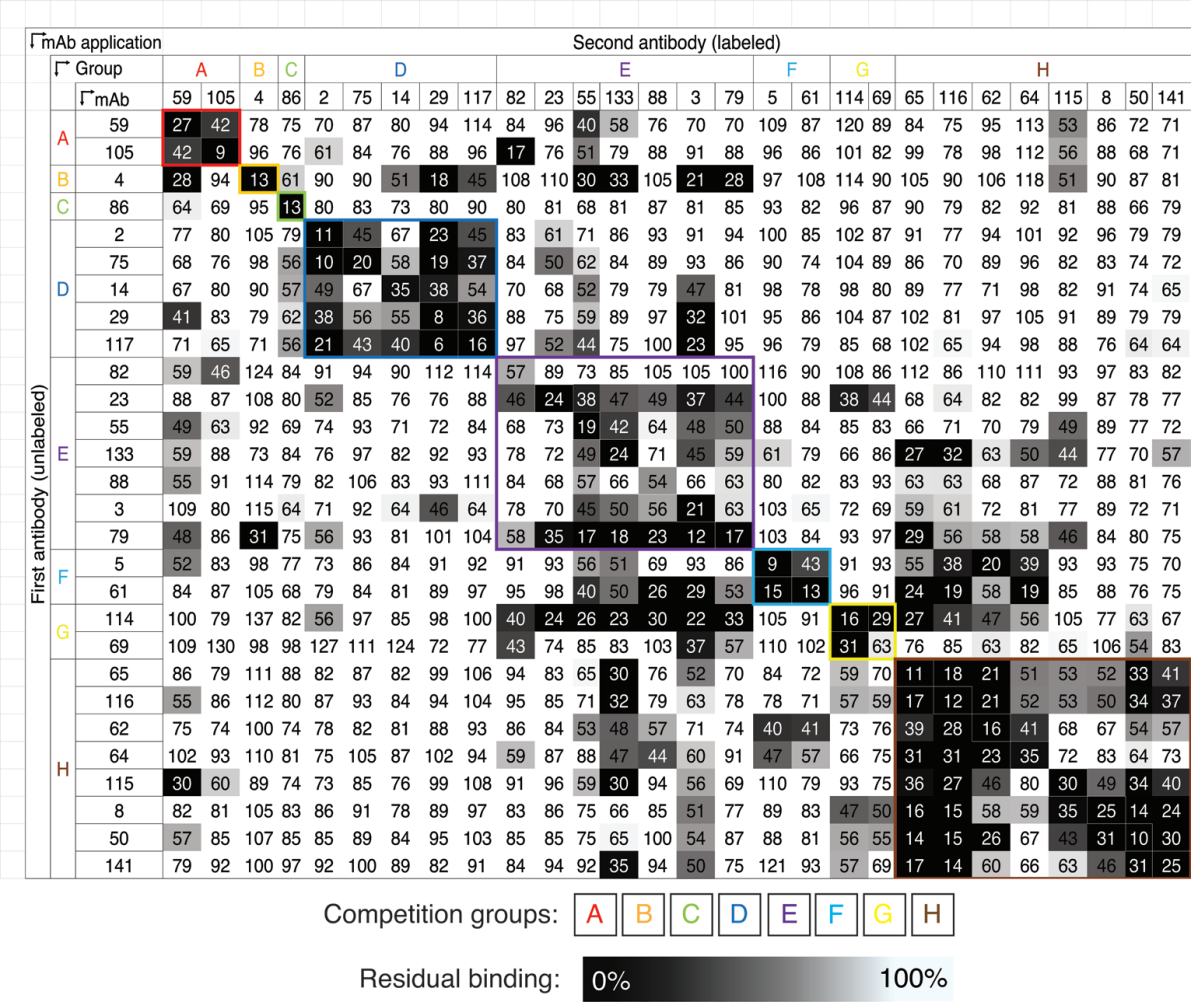
Competition assessment of human antibodies to CCHFV using M segment–expressing cells. We tested 28 mAbs in competition-binding assays. The mAbs are displayed in 8 groups (designated A–H) based on their ability to compete for binding with each other. The values shown are the percentage of binding that occurred during competition compared with noncompeted binding of the mAb. This value was normalized to 100%. The values are also indicated by the box fill color; darker colors toward black indicate higher competition, and lighter colors toward white indicate less competition, on a gradient scale. Values shown are the average of 2 biologically independent experiments with triplicate values in each experiment.

mAb immunoglobulin proteins were expressed, purified, and tested for binding specificity and function. We categorized the specificity of mAbs from the entire panel based on the results of binding assays with the purified antibody from hybridomas to antigen in cells transiently expressing the M segment of IbAr10200 and/or the Gc protein (Supplemental Figure 1). Most of the clones isolated bound to Gc, as was indicated by cell display constructs expressing either the M segment or Gc (Supplemental Figure 1). The signal magnitude of Gc binding over M segment binding may be driven by a degree of masking of sites on the Gc protein by the presence of MLD/GP38/Gn in the M segment construct versus the exposure of those sites in the Gc-only construct. Within the full panel, only 6 of the antibodies bound to the M segment but not to the Gc-only expressing cells (Supplemental Figure 2). Curves for binding of the mAbs from the first half of the panel binding to cell surface–displayed M segment indicate a range of strong and weak binding (Supplemental Figure 3). Using mAbs from the first half of the panel in competition-binding assays on M segment–transfected cells, we observed roughly 8 competition groups (Figure 2).

**Figure 3:**
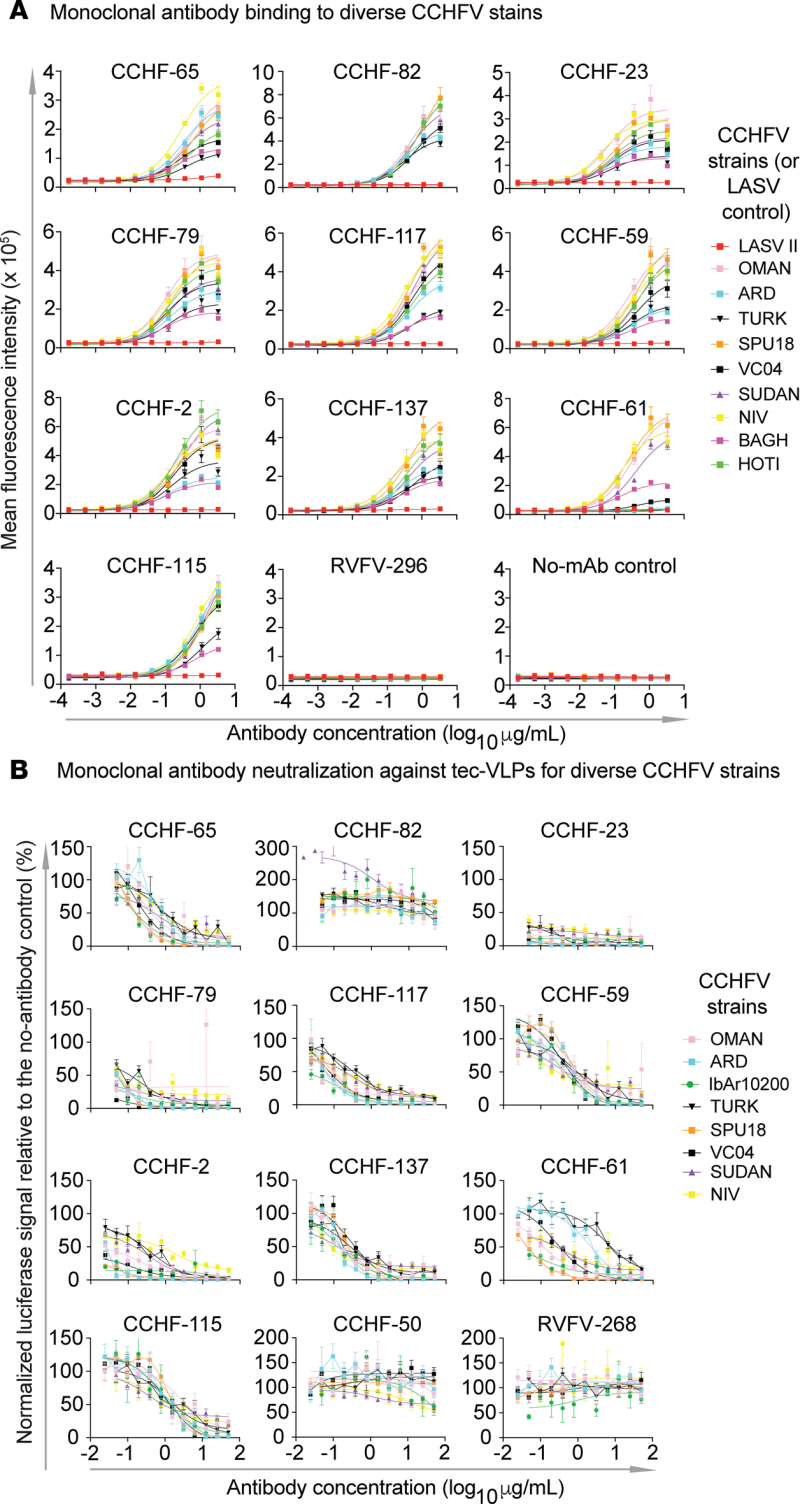
CCHFV-specific mAbs bind cells expressing the M segment of various strains of CCHFV with varying capacities and neutralize tecVLPs expressing Gc/Gn from various strains of CCHFV. (**A**) A binding titration was performed with suspension cells expressing the M segment of various strains of CCHFV. The assay was performed in biological triplicate with technical duplicates. (**B**) A neutralization assay was performed with tecVLPs on BHK21-T7 cells with mAbs that bound to either Gc or GP38. Hybridoma-derived mAbs were prepared and diluted serially before mixing with approximately 5,000 luciferase units of tecVLP. After 1 hour incubation at 37°C, the mixture was added to a BHK21-T7 cell monolayer, and cells were allowed to incubate prior to lysis and processing. The assay was performed in biological triplicate and technical duplicates with mAb RVFV-268 included as the mAb negative control. Data were analyzed using a 3-parameter, nonlinear fit analysis in Prism software version 9 (GraphPad). Data represent mean ± SEM.

Next, we tested the antibodies from the entire panel for neutralizing activity using transcription and entry-competent virus-like particles (tecVLPs) based on the sequence of CCHFV strain IbAr10200. These particles contain all CCHFV proteins and a minigenome that is morphologically consistent with authentic CCHFV. The mAbs were tested against the tecVLPs in a 4-point concentration neutralization assay. Most neutralizing activity was observed in antibodies that target Gc, while the antibodies that did not bind Gc either did not neutralize or they enhanced infection of the IbAr10200 strain tecVLP (Supplemental Figure 4). Next, we examined representative neutralizing and nonneutralizing mAbs from the first half of the panel from competition groups A, D, E, F, H, and CCHF-137 for breadth of binding (cross-reactive recognition of antigens based on the sequences of diverse field strains of CCHFV). In this experiment, each of the antibodies tested bound to Gc with the exception of CCHF-82 and CCHF-65 (target not determined). We performed binding assays using titrated antibodies on antigens from various strains of CCHFV using the M segment–transfected methods for cell display of GPC containing Gc, Gn, and GP38; Lassa fever virus II antigens were used as a control for nonspecific binding. We observed that most of the mAbs cross-reactively bound to diverse CCHFV strains in the panel (Figure 3A and Supplemental Table 2).

### Cross-neutralizing human antibodies to diverse strains of CCHFV identified using the tecVLP system.

**Figure 4:**
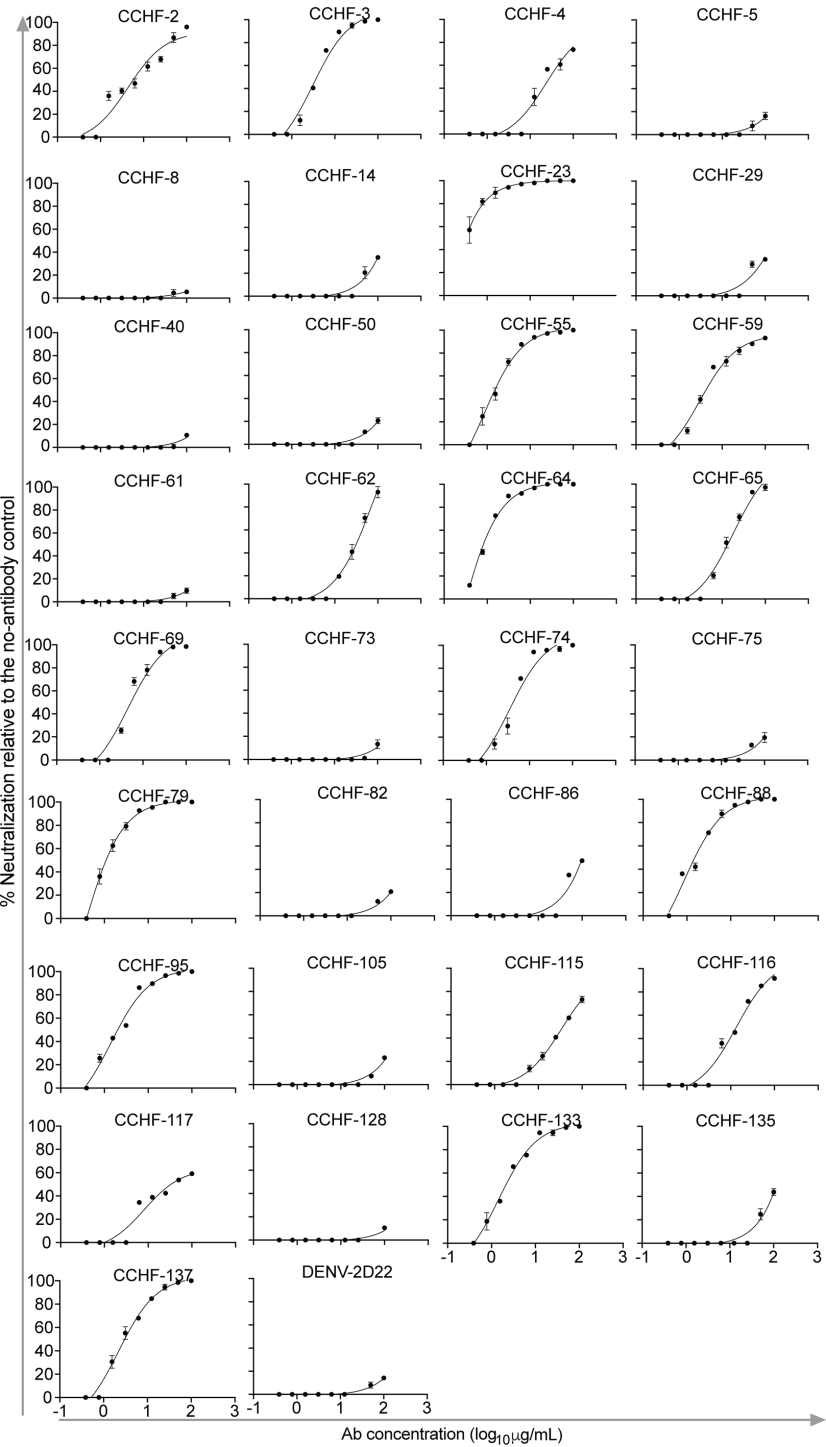
CCHFV antibodies neutralize the authentic IbAr10200 strain of CCHFV. CCHFV-specific mAbs were serially titrated and mixed with authentic CCHFV IbAr10200 strain. Antibody and virus mixture was added to SW-13 cells. Plaques were counted, and percent neutralization was reported relative to the no antibody control. The assay was performed once with technical duplicates. Data were analyzed using a log (inhibitor) versus response (3-parameter) nonlinear fit analysis in Prism software version 9 (GraphPad). Data represent mean ± SEM.

We then asked whether representative neutralizing and nonneutralizing mAbs from the first half of the panel from competition groups A, D, E, F, H, and CCHF-137 that cross-reactively bind to antigens from diverse strains of CCHFV can also neutralize diverse virus strains on the tecVLP system. We serially diluted the 11 broadly binding antibodies and performed neutralization assays with the luciferase reporter system. In this experiment, all antibodies bound to Gc except CCHF-82 and CCHF-65 (target not determined). We observed that all antibodies except CCHF-82, CCHF-50, and the negative control antibody RVFV-296 neutralized diverse CCHFV strains, with a range of potencies noted (Figure 3B and Supplemental Table 3). We then tested the first half of the antibody panel for neutralization of authentic CCHFV strain IbAr10200 (Figure 4). mAb CCHF-23 was the most potently neutralizing antibody we isolated against both the strain IbAr10200 tecVLP and the authentic IbAr10200 virus. We then further titrated the most potent antibodies from the entire panel against the tecVLPs based on the IbAr10200 and Sudan strains to assess the potential to recognize multiple genotype III strains. We observed potent neutralization with the 4 Gc-targeting mAbs, CCHF-23, -196, -245, and -263, against the tecVLP expressing the IbAr10200 or Sudan complete GPC (Supplemental Figure 5). Additional testing of the mAbs from the second half of the panel against authentic virus is of interest for future studies. Of the Gc-specific antibodies, CCHF-23 is perhaps the most promising for development as a medical countermeasure because of its potency and breadth of neutralization. Testing of a panel of CCHFV mAbs against the authentic strain IbAr10200 showed most antibodies to this target are only modestly neutralizing, and potently neutralizing antibodies are rare (Figure 4). Unfortunately, we were unable to convert CCHF-23 from hybridoma-cell-based expression to a recombinant format with efficient expression, most likely due to multiple predicted sequence liabilities (deamidation sites) in the complementarity-determining regions that may have been differentially modified between the hybridoma cells (mouse-human hybrid) and Expi293F cells. Further antibody engineering work may be needed to optimize the expression of this antibody at high concentrations in a manufacturing setting.

### PreGn binding human mAbs compete for binding with reference murine mAbs.

**Figure 5:**
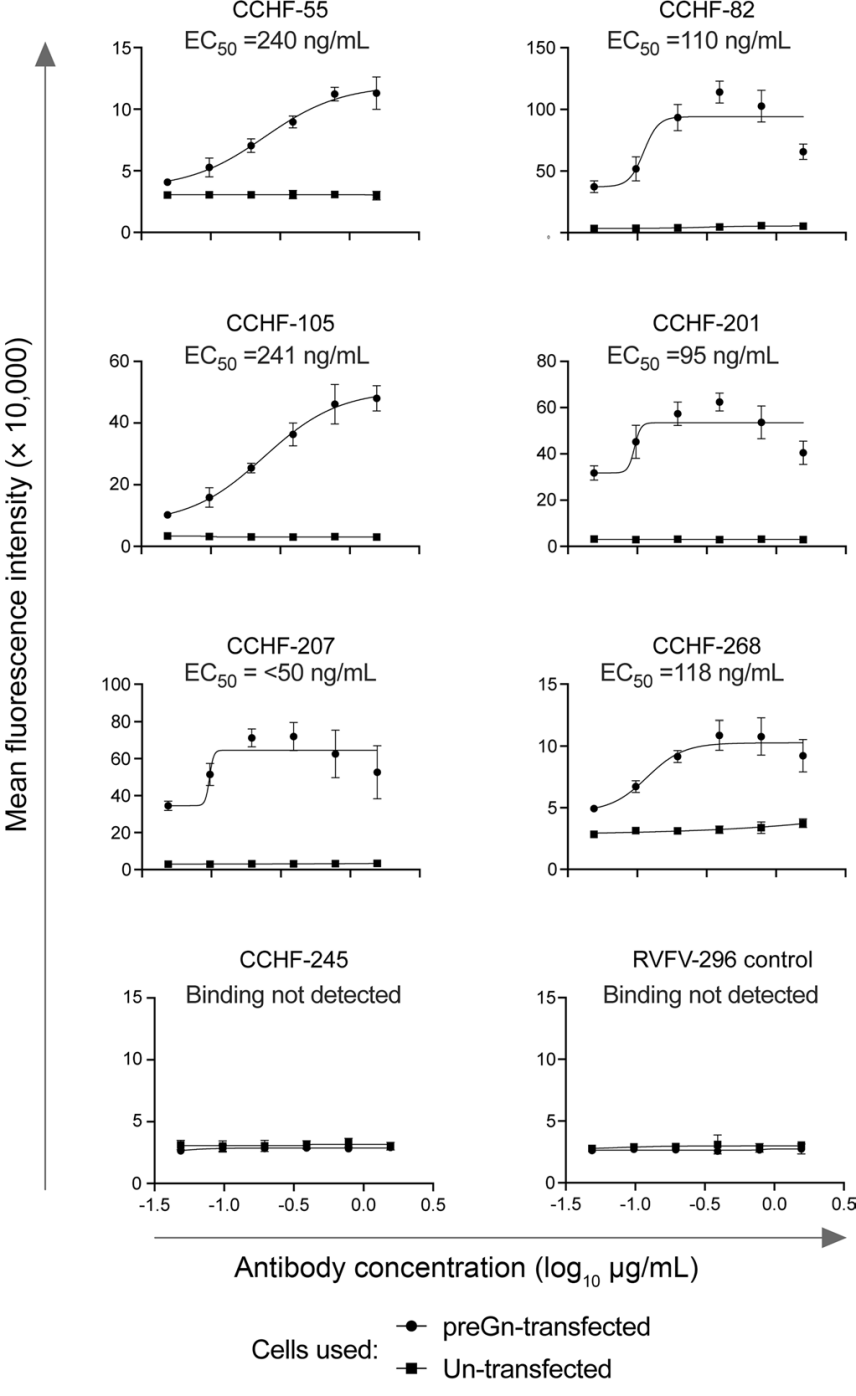
PreGn-identifying antibodies bind to preGn-expressing cells. PreGn binding antibodies were titrated and further assessed for their capacity to bind preGn-expressing cells. mAbs were serially titrated and allowed to incubate on preGn transiently expressing cells before washing and adding PE-conjugated secondary antibody. Secondary antibody was washed, and cells were resuspended for analysis on an iQue flow cytometer. Binding to transfected cells was observed relative to untransfected cells, and data are presented as mean values ± SD and represent duplicate values from each of 3 independent experiments. IC_50_ values were calculated using a 4-parameter sigmoidal nonlinear fit in Prism software version 9 (GraphPad). CCHF-245 was used as a negative control.

**Figure 6:**
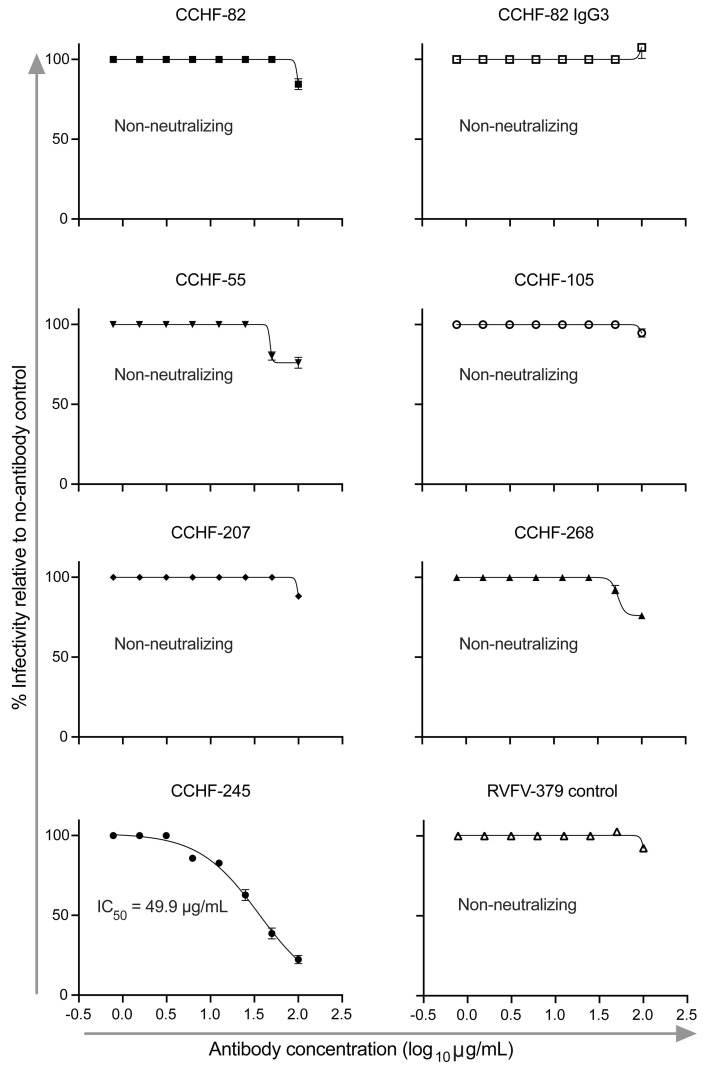
PreGn binding antibodies do not neutralize WT Turkey 200406546 strain of CCHFV. The preGn binding antibodies were serially diluted and tested against the Turkey 200406546 strain of CCHFV in a BSL-4 neutralization assay. Briefly, antibody and virus were premixed, added to a SW-13 monolayer, overlayed with 1.25% Avicel, and incubated for 4 days. Cells were fixed in formalin, stained with 1% crystal violet, and enumerated. Data are presented as mean ± SD and represent duplicate values from a single experiment. IC_50_ values were calculated using a Sigmoidal 4-parameter, nonlinear fit in Prism software version 9 (GraphPad). CCHF-245 was used as a positive control.

**Figure 7:**
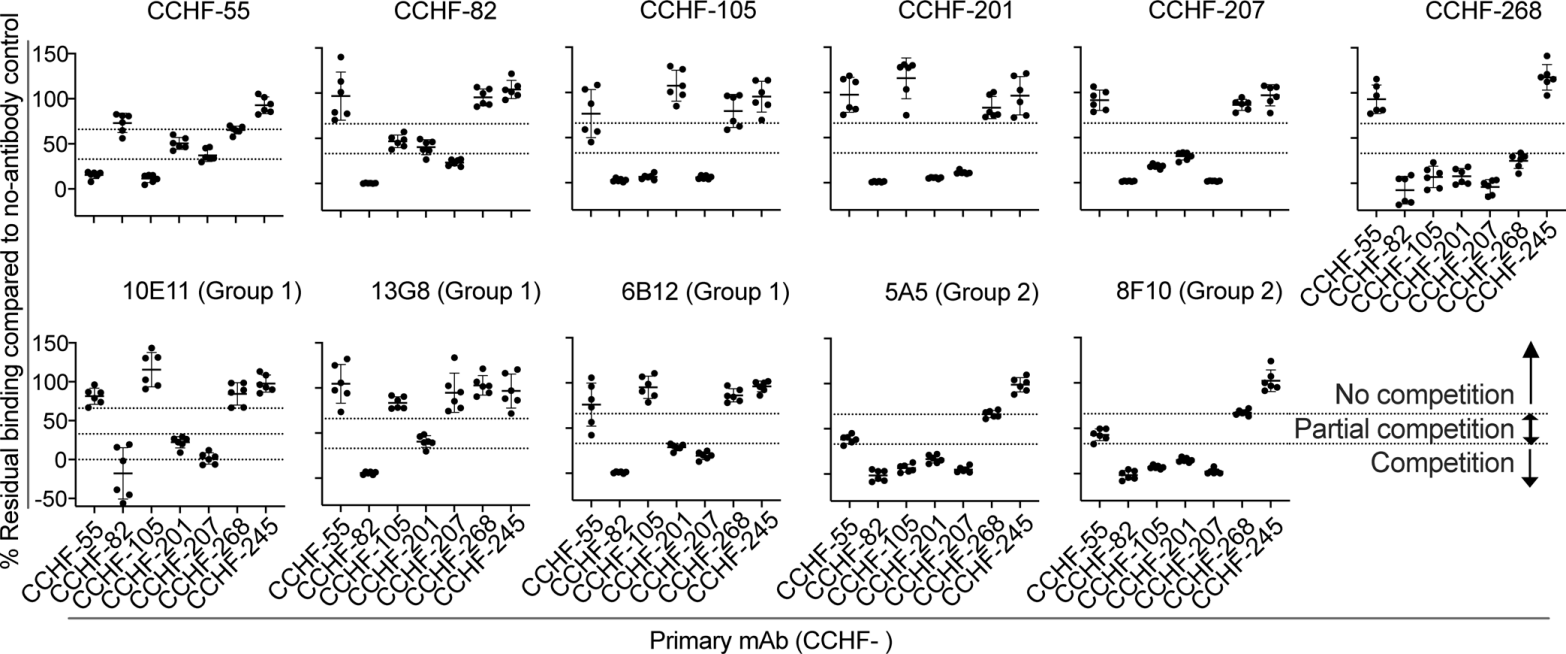
PreGn-identifying antibodies compete with reference mouse monoclonal antibodies to GP38 and other preGn binding human antibodies. PreGn binding antibodies were added to preGn-expressing cells at saturating concentrations (20 μg/mL) for 30 minutes. Alexa Fluor 647–conjugated mouse or human antibodies were then added to cells at 5 μg/mL without a prior wash step. Cells were washed and immediately processed on an iQue flow cytometer. The values shown are the percentage of binding that occurred during competition compared with noncompeted binding of the mAb. This value was normalized to 100%. The values are also indicated by the dotted lines; above 40% indicated no competition, between 40% and 20% indicated partial competition, and below 20% indicated competition. Values shown are from 2 independent experiments with 3 replicates. Data represent mean ± SD.

We next sought to organize the antibodies into phenotypically consistent groups that recognize similar major antigenic sites by testing whether any of the 6 mAbs that bound to the M segment but not to the Gc protein recognized preGn protein (Figure 5). One of these preGn mAbs, CCHF-82, showed some enhancement of infection in the tecVLP system, particularly for the IbAr10200 strain tecVLP (Figure 3B). This finding is potentially an artifact of the NanoLuc-based neutralization assay or is due to the antigenic difference in the tecVLP construct where GP38 may not be properly cleaved from the VLP. This enhancement was not observed with the WT virus. Notably, enhancement was not observed for CCHF-82 against authentic virus testing with the IbAr10200 strain (Figure 4). We then tested these mAbs against the Turkey 200406546 strain with the intent to move toward testing a candidate in an animal study. These mAbs did not neutralize the authentic virus Turkish strain of CCHFV as similarly reflected by 4 other preGn-binding antibodies from the full panel (Figure 6). We next sought to determine if any of these preGn mAbs could compete with known GP38-reactive murine mAbs for binding to preGn (Figure 7). Using the preGn display system, we tested murine antibodies for binding in the presence of saturating concentrations of the human antibodies. Groups were defined using the grouping nomenclature used by Golden et al. (19). Human mAb CCHF-105 competed only with group 2 mouse antibodies, while CCHF-82 competed or partially competed with both groups 1 and 2 antibodies. Notably, CCHF-82 competed with the mouse antibody 13G8, a nonneutralizing mAb that exhibited protection in a mouse model of infection (19).

### In vivo testing of 5 human mAbs.

**Figure 8:**
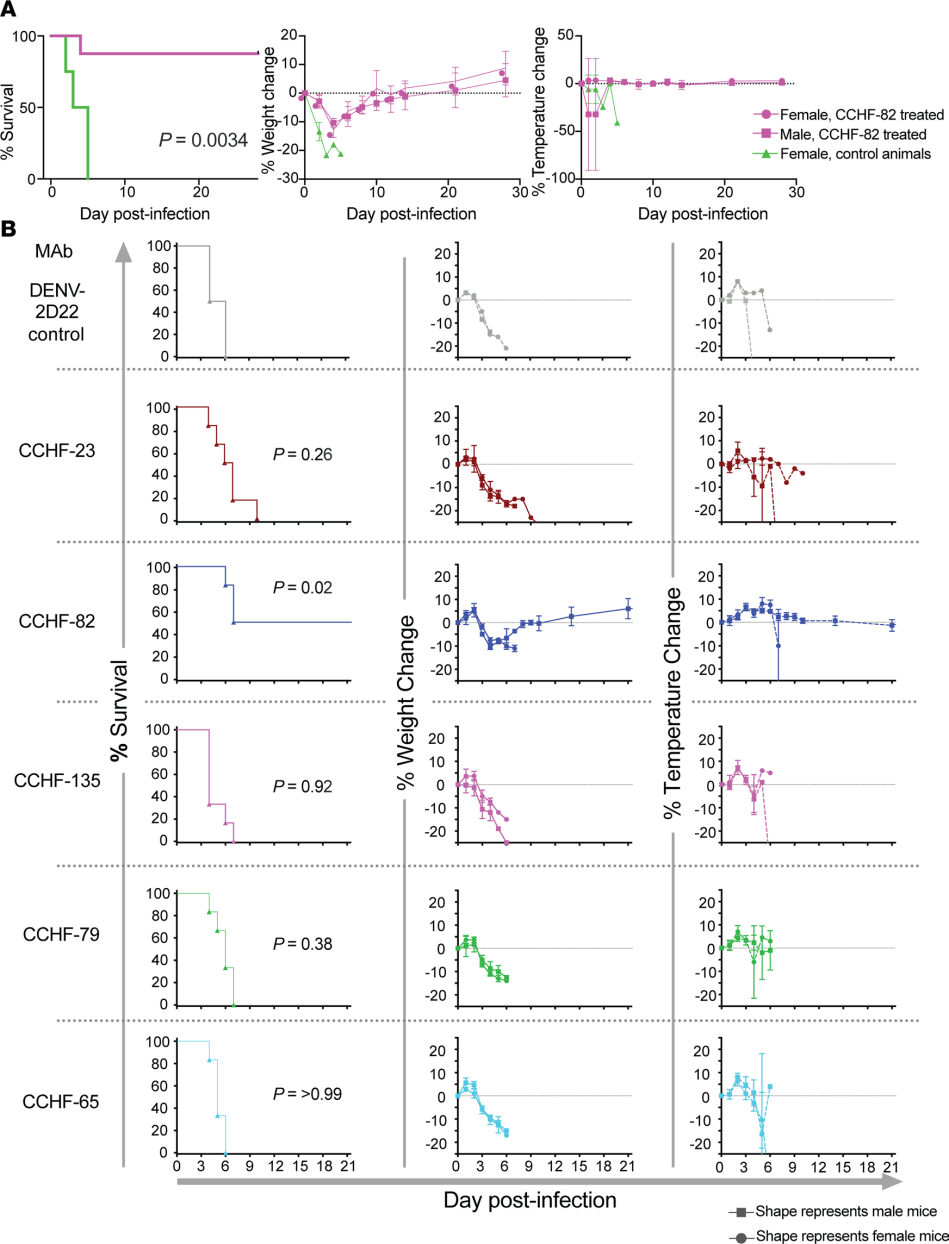
CCHF-82 provides partial protection as a postexposure prophylaxis against the Turkish strain of CCHFV in the STAT1-KO mouse model. (**A**) Prophylaxis study: mAbs were administered once by the i.p. route to STAT1-KO mice (*n* = 8 CCHF-82 treated animals; *n* = 4 control animals). Animals were challenged 24 hours later with 100 PFUs of CCHFV Turkey 200406546 strain. Kaplan-Meier survival curves were statistically analyzed using a log-rank (Mantel-Cox) test where treated animals (*P* values shown in figure) were compared with animals treated as negative control. Weight and temperature graphs reflect group means ± SD of the percent change in weight or temperature of animals relative to the weight or temperature the day of virus challenge. *P* values for each condition tested compared with the control group using a log-rank (Mantel-Cox) test. (**B**) Postexposure prophylaxis study: a single dose of mAb was administered by the i.p. route to STAT1-KO mice (*n* = 6 per group; 3 male and 3 female) at 30 minutes after infection. 100 PFUs of CCHFV Turkey 200406546 strain was administered by the i.p. route. CCHF-23, CCHF-135, CCHF-65, CCHF-82, CCHF-79, and DENV-2D22 (an isotype-matched negative control mAb) were tested in a single dose of 250 μg per mouse except for CCHFV-135 at 125 μg per mouse. In weight and temperature curves, results from male mice are designated by circles and those from female mice by squares. Kaplan-Meier survival curves were statistically analyzed using a log-rank (Mantel-Cox) test where mAb-treated animals (*P* value shown in figure) were compared with animals treated with the DENV-2D22 negative control mAb using Prism 9 software (GraphPad). Weight and temperature data represent mean ± SD as a percentage of their weight and temperature the day of the virus challenge.

**Figure 9:**
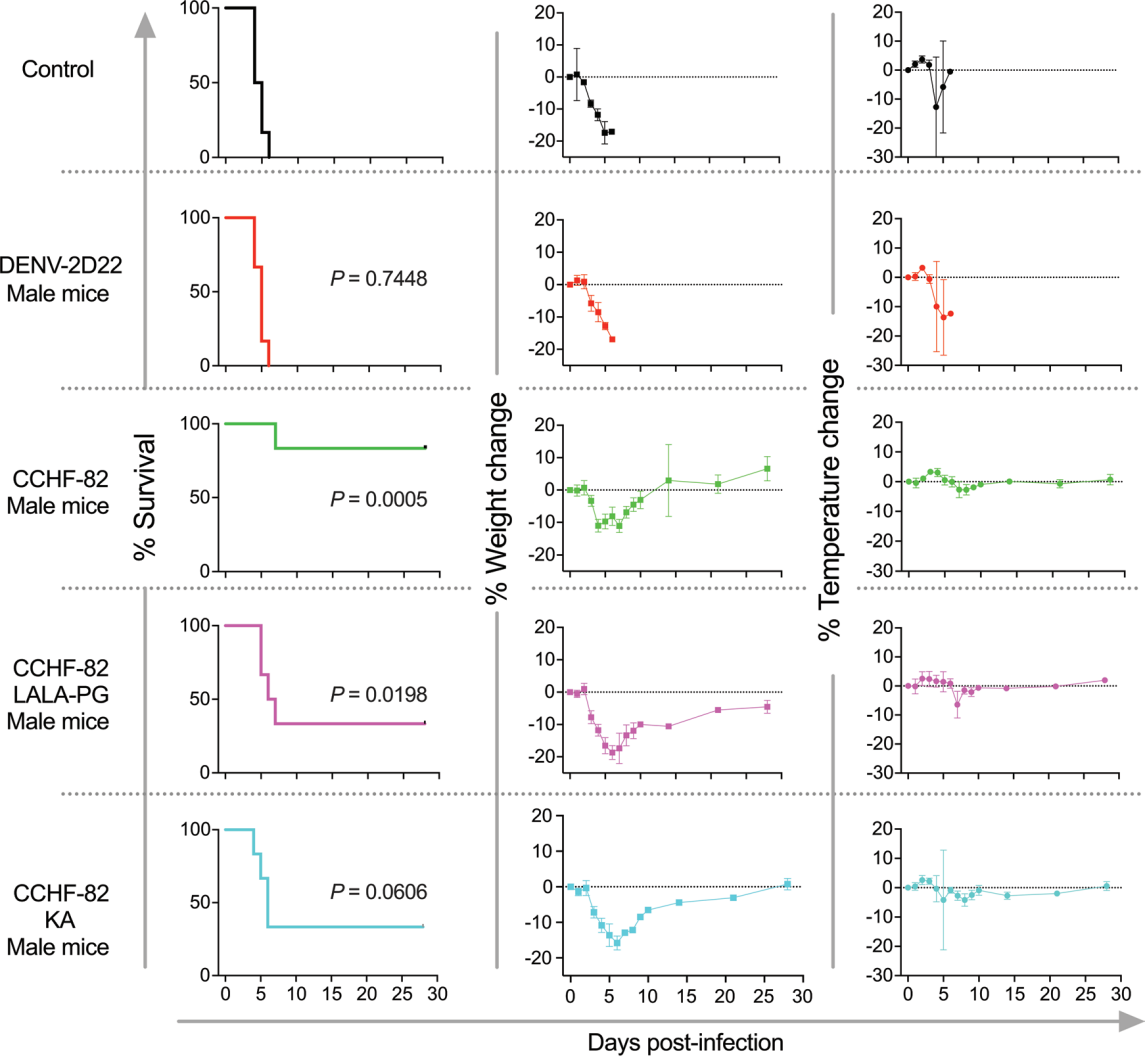
CCHF-82 protection capacity is primarily driven by complement activation as a postexposure prophylaxis against the Turkish strain of CCHFV in the STAT1-KO mouse model. mAbs were administered once by the i.p. route to STAT1-KO mice (*n* = 6 per group). Animals were challenged 24 hours later with 100 PFUs of CCHFV Turkey 200406546 strain. Kaplan-Meier survival curves were statistically analyzed using a log-rank (Mantel-Cox) test where treated animals (*P* values shown in figure) were compared with animals treated as negative control. Weight and temperature graphs reflect group mean ± SD of the percent change in weight or temperature of animals relative to the weight or temperature of the day of virus challenge. *P* values were generated for each condition tested compared with the control group using a log-rank (Mantel-Cox) test using Prism 9 software (GraphPad).

Given the ability of CCHF-82 to directly compete with the known protective mouse antibody 13G8, we sought to determine whether a recombinant IgG1 form of human mAb CCHF-82 protects against CCHFV challenge by performing a series of mouse experiments. We performed a virus challenge study with prophylactic administration of mAb. Using CCHFV Turkey 200406546 strain in the STAT1-knockout mouse model, CCHF-82 IgG1 provided a significant level of protection (*P* = 0.0034) relative to control-treated animals (Figure 8A and Supplemental Figure 6A). In the same mouse and virus model, we next compared the effect of this nonneutralizing mAb CCHF-82 with several of the most potently neutralizing antibodies in a postexposure administration study design. For this experiment, we chose 3 neutralizing mAbs (CCHF-65, CCHF-23, and CCHF-79) and 2 nonneutralizing mAbs (CCHF-82 and CCHF-135) from the first half of the antibody panel to capture diverse patterns of neutralizing and nonneutralizing antibodies from competition groups E and H, along with CCHF-135 (not tested in the competition assay). The lot tested for CCHF-23 IgG purified in this experiment was from hybridoma cell expression given its high degree of neutralization to IbAr10200. We did not observe protection with any of the neutralizing antibodies we tested against the CCHFV Turkey 200406546 strain. We observed 50% protection with CCHF-82, the nonneutralizing GP38 recognizing antibody (*P* = 0.0193), which is comparable to the best-in-class GP38 targeting mAbs reported to date (23, 33) (Figure 8B and Supplemental Figure 6B). We noted a sex-based difference in the outcome of the postexposure study, in that the female mice treated with CCHF-82 survived, whereas the male mice did not (Supplemental Figure 6B). We further probed the mechanistic basis of the protective capacity of CCHF-82 by mutating the Fc domain of the mAb to abrogate Fc effector function with L234A/L235A/P329G mutations in the Fc region (LALA-PG) or knocking down complement activity with a K322A mutation in the Fc region (KA). We observed that the intact antibody provided a significant level of protection, as expected in this study against the CCHFV Turkey 200406546 strain in the STAT1-KO male mice in a prophylactic study design (*P* = 0.0005). The LALA-PG Fc variant IgG provided a modest level of protection, with 2 of 6 animals surviving (*P* = 0.0198), and the KA Fc variant IgG provided an insignificant level of protection, with 2 of 6 animals surviving (*P* = 0.0606). The weights and temperature curves indicate a trend toward faster recovery with the intact antibody versus LALA-PG or KA variant IgGs (Figure 9 and Supplemental Figure 6C). The impact of sex difference in the context of mAb prophylaxis was not as clear as in the postexposure prophylactic study but deserves further investigation.

## Discussion

Protection against infection or disease after prior exposure to most bunyavirus infections or vaccinations typically correlates well with the serum concentration of neutralizing antibodies to surface proteins, but the mechanisms of immunity to CCHFV seem to contradict this principle (19–23, 26–33, 35). Here, we studied in detail the dominant features in the B cell response of a human survivor to CCHFV infection soon after recovery. We observed diverse patterns of molecular recognition of CCHFV by mAbs encoded in the circulating B cell repertoire, and the clones induced by infection exhibited use of diverse antibody variable genes. Notably, a complex pattern of recognition was demonstrated by the competition-binding patterns in which some groups showed substantial overlap with others. This overlap could indicate that some epitopes may span the footprint of more than 1 major antigenic site; alternatively, the Fc regions of bound antibodies recognizing differing but adjacent sites may cause steric hindrance. Although we isolated Gc-specific neutralizing antibodies that cross-reacted to many of the CCHFV strains we tested, the potency of these mAbs was modest. We did observe the induction of a portion of B cells encoding nonneutralizing antibodies that recognized the viral GP38 protein, which was recently suggested to possess toxin-like activities (20). Antibodies targeting GP38 provided some protection against CCHFV infection as a postexposure prophylactic in a lethal mouse model of infection against the Turkish strain. Thus, in the human survivor of the CCHFV infection we studied, the responding B cell repertoire principally encoded Gc-specific neutralizing antibodies and GP38-binding antibodies that may offer some degree of protection against CCHF infection. Limited cross-clade protection with mAbs targeting either Gc or GP38 has been observed (17, 23, 35). Here, we aimed to assess the human antibody response in a donor who was infected with a strain of CCHFV from genotype III (Africa 3) (5, 36). Understanding cross-clade protection by human antibodies of strains from geographic regions that experience sporadic outbreaks of different CCHFV genotypes may be informative for planning public health measures (37).

The most interesting antibody we isolated here is perhaps the clone CCHF-82, which competed for binding with competition groups 1 and 2 murine GP38-reactive antibodies from a previous report (19). CCHF-82 conferred a significant degree of protection in the STAT1-deficient mouse model of CCHFV infection. We observed male mice succumbing despite antibody treatment in the second animal study in which the mAb treatment was given as a postexposure prophylactic rather than as a preexposure prophylactic. It is interesting that this minor difference in treatment timing made it more difficult to protect the male mice compared with the female mice. It has been reported that male mice are more susceptible to severe disease following challenge with the mouse-adapted Hoti strain CCHFV (38, 39). It is unclear the degree to which male susceptibility played a role in our animal studies using the CCHFV Turkey 200406546 strain in STAT1-KO mice with relation to antibody treatment.

We were surprised by the significant level of protection observed, given the antigenic distance of the challenge strain from the infecting strain (a close relative to ArD39554) in the GP38 protein. The pairwise amino acid identity of the GP38 protein (amino acids 248–515 of IbAr10200 M segment) between Ard39554 (DQ211628.1) and IbAr10200 (NC_005300.2) is 71.6%, while the pairwise amino acid identity of the GP38 protein between ArD39554 and CCHFV Turkey 200406546 (KY362519.1) strains is 70.9%. The pairwise amino acid identity of the GP38 protein between IbAr10200 and CCHFV Turkey 200406546 is 84%. Given the low level of GP38 homology (70.9%) between the ArD39554 and CCHFV Turkey 200406546 strains, the level of protection in this stringent mouse model is notable. Further investigation with this antibody in prophylaxis and therapeutic models with the CCHFV strain IbAr10200 would be of interest in the future, since the GP38 sequence of that strain also differs substantially from that of the ArD39554 strain.

Nonneutralizing mouse monoclonal antibodies have proven protective in certain mouse models of CCHFV infection (19, 33). Murine mAb 13G8 binds to GP38 and does not neutralize CCHFV (19). The mAb 13G8 protected against lethal challenge when administered as a prophylactic or a therapeutic agent against experimental CCHFV challenge (19). Here, we observed the presence of circulating B cells that encode GP38-recognizing antibodies, although they were present at lower frequency than Gc-binding memory B cells in this survivor. A recent study of GP38 suggested the protein has properties similar to a viral-associated, toxin-like dengue virus protein, nonstructural 1, with associated vascular leak and endothelial barrier dysfunction (20). If GP38 contributes to pathogenesis in this way, then antibodies such as CCHF-82 may provide protection against CCHF infection or disease through antitoxin activities (rather than through classical virus neutralization). Moreover, previous studies indicate murine mAb 13G8 functions in part by fixing complement, which contributes to clearance of viruses or virus-infected cells. The role of bound Fc molecules in enhancement complement deposition during CCHFV should be clarified. It may be that early in infection complement contributes to protection; however, we caution that enhanced Fc-mediated complement deposition in tissues during the later stages of a highly inflammatory acute viral infection later might exacerbate disease. Our data provide evidence that the human mAb CCHF-82 protects in STAT1^–/–^ mice against CCHFV Turkey 200406546 strain via complement-mediated activity. We observed a drop in protective efficacy when all Fc effector functions were ablated or when complement activity was diminished. Interestingly, the LALA-PG and KA variant IgG forms of CCHF-82 yielded similar survival curves. These data suggest that complement activity of CCHF-82 contributed substantially to the overall protective capacity of CCHF-82 in murine challenge with CCHFV Turkey 200406546 strain. It appeared that although 2 of 6 male mice treated with the KA variant IgG form of CCHF-82 survived, this finding was not significant, and further experimentation needs to be done to understand the degree to which toxin-blocking activity of this particular antibody is important in C3^–/–^ mice. The degree of complement-meditated activity, Fc effector function, and toxin-blocking activity in the overall protective capacity of a GP38-targeting mAb may be driven by a constellation of effects mediated by epitope recognition patterns, antibody isotype, and subclass. To fully understand the complexity of these interactions, further investigation is warranted with additional isolation and study of human antibodies. Additionally, the protective capacity of human antibodies directed toward NP has not been systematically assessed despite evidence of their protective capacity in mice following vaccination and using the murine mAb 9D5 (30, 31, 40).

It is surprising that Gc-specific antibodies we and others have isolated are not more potently neutralizing. It is possible the sites of most vulnerability to neutralization are poorly accessible and that antibodies recognizing those sites are subdominant. The LDL receptor has recently been described as a receptor for CCHFV (41–43). Additionally, the use of cellular membrane proteins in the virion particle that may mediate entry into the cell may complicate a simple glycoprotein-mediated neutralization model of protection, such as the use of apoE (43). We reason that blocking receptor engagement ought to neutralize virus infection efficiently. More work to determine the receptor binding domain is required and to see if neutralizing antibodies can block receptor binding on the Gc protein as an avenue for rational antibody combination design.

Although we attempted to identify antibodies that bound to either Gn or the interface between Gc/Gn, none were identified. This finding does not necessarily mean that these antibodies do not exist but rather suggests that they are rare in this particular survivor or that the approaches used for Gn antibody discovery thus far are not able to sufficiently identify them. We observed in sera the existence of CCHFV Gn-specific antibodies (Supplemental Figure 7). Alternative approaches for Gn antibody may be warranted using Gc/Gn stabilized immunogen design (44), virus-like particles, or a functional neutralization screening test to aid in the discovery of antibodies recognizing epitopes for improved neutralization potency and their associated B cells.

## Methods

### Sex as a biological variable.

Both male and female animals were studied, and the effect of each sex on the outcome was taken into consideration. The human blood sample studies from which the human mAbs were derived were based on the response of a single female survivor of infection.

### Human blood samples.

Human PBMCs were obtained from a survivor of natural infection with a strain of CCHFV from genotype III (5, 37). First, PBMCs were collected from the individual a few months after the illness had resolved, following informed written consent through an approved program maintained by the Instituto de Salud Carlos III. Second, serum samples were obtained in Turkey, following informed written consent from convalescent patients. The studies were approved by the Institutional Review Boards of Vanderbilt University Medical Center and the ethics and research committees of Hospital Universitario La Paz–Carlos III–Cantoblanco in Madrid and Maramara University in Istanbul, Turkey.

### Cell culture.

SW-13 (American Type Culture Collection [ATCC] CCL-105) (female, adrenal gland/cortex) cells were used for WT virus neutralization assays in BSL-4 containment. SW-13 cell cultures were maintained at 37°C in Leibovitz’s L-15 Medium (ATCC, catalog 30-2008) and were supplemented with FBS to a final concentration of 10% with a free gas exchange with atmospheric air. BHK-21 (clone BSR T7/5) cell lines were a gift from U. Buchholz (National Institute of Allergy and Infectious Diseases, Bethesda, Maryland, USA). Briefly, this cell line was generated by transfection with pSC6-T7-NEO, encoding the T7 RNA polymerase gene under control of the cytomegalovirus promoter and the neomycin resistance gene (the plasmid was a gift from M. Billeter, University of Zurich, Zurich, Switzerland). BHK-21 cells were maintained at 37°C in 5% CO_2_ in DMEM (Thermo Fisher Scientific) containing 10% (v/v) heat-inactivated FBS (HyClone), 10 mM HEPES pH 7.3, 1 mM sodium pyruvate, 1′ nonessential amino acids, and 100 U mL of penicillin-streptomycin with geneticin selection every other passage. Expi293F cells (Thermo Fisher Scientific, A1452) were maintained at 37 °C in 8% CO_2_ in Expi293F Expression Medium (Thermo Fisher Scientific, A1435102). The HMMA 2.5 nonsecreting mouse-human heteromyeloma cell line (female mouse and female human) was provided by L. Cavacini and M. Posner (Dana-Farber Cancer Institute, Boston, Massachusetts, USA) and cultured as previously described (45, 46). Mycoplasma testing of Expi293F and BHK-21 cultures was performed on a monthly and bimonthly basis, respectively, using a PCR-based mycoplasma detection kit (ATCC, catalog 30-1012 K), and all tests were negative during the time of study.

### Viruses and tecVLPs.

For production of tecVLPs, we used a recombinant plasmid system as previously described (24, 25). The vector pCAGGS (pC-GPC) was used to express the following ORFs (codon optimized and synthesized by GenScript) of the GPC of CCHFV isolates: ArD15786 (DQ211627), Baghdad-12 (AJ538197), Kosova Hoti (EU037902), NIV112143 (JN572085), SPU18/88 (KJ682810), Sudan Al-Fulah 3–2008 (HQ378185), YL04057 (FJ562094), IbAr10200, Oman199809166, Turkey200406546, and Afg09 (25). CCHFV helper plasmids, also previously described (24, 25), encoding the strain IbAr10200 NP (pC-NP), the codon-optimized L (pCLCK-L, possessing an R substitution at position 16) helper plasmids, and the pL-Luc minigenome plasmids were used. BHK-21 cells stably expressing the T7 RNA polymerase were transfected with pC-NP, pCLCK-L, pL-Luc, and a pC-GPC plasmid using TransIT-LT1 Transfection Reagent according to the manufacturer’s recommendations (Mirus Bio). Media was replaced with fresh culture medium the following day, and tecVLP-containing cell supernatants were collected. Virus was passaged and titrated as previously described (25).

CCHFV isolates Turkey-812955 (KY362515, KY362517, and KY362519) and IbAr10200 were propagated in SW-13 cells. The CCHFV IbAr10200 and CCHFV Turkey 200406546 strains were passaged and titrated by plaque assay in SW-13 cells. The work was approved by The University of Texas Medical Branch (UTMB) Institutional Biosafety Committees and conducted in approved BSL-4 facilities. For the animal efficacy studies, we used CCHFV Turkey 200406546 strain.

### Mouse models of infection.

Adult (STAT1-KO) mice were obtained from Taconic Biosciences. Mice were housed in microisolator cages and provided water and food ad libitum. The mouse CCHFV challenge efficacy studies were approved by the UTMB Institutional Biosafety Committees and conducted in Select Agent–approved animal BSL-4 facilities.

### Virus neutralization assay.

TecVLP generation in BHK-21 T7-expressing cells and neutralization assays were conducted as described previously (24, 25). Briefly, NanoLuc signal was assessed in BHK-21 cells incubated with the tecVLPs of various strains indicated above. Culture media was removed from cells, and the cells were washed with PBS and incubated for 45 minutes in passive lysis buffer (Promega) at room temperature. Cell lysate (20 μL) was removed and added to 20 μL of Nano-Glo Luciferase Assay System (Promega) to detect NanoLuc signal in opaque white 96-well plates using CLARIOstar by BMG LABTECH. Data were normalized to background signal and expressed as a percentage of no virus control.

For the CCHFV plaque reduction neutralization test, mAbs were serially diluted in SW-13 media and mixed with equal volumes of SW-13 medium containing CCHFV viral particles (100 PFUs per well), and this mixture was incubated for 1 hour at 37°C. Each suspension was added to a monolayer of SW-13 cells of a 6-well plate for 1 hour at 37°C. Either CCHFV IbAr10200 or Turkey 200406546 strain was used as indicated in respective assays. Following incubation, wells were overlayed with Avicel and incubated for 3 days in 5% CO_2_ at 37°C. Following incubation, overlay was removed, and cells were fixed and stained for 1 hour with formalin containing crystal violet. Plaques were enumerated by visual examination, and reductions of plaque number were reported. The percent relative infection was determined based on the virus-only control. Tests were performed for each antibody in triplicate. IC_50_ values were determined using a sigmoidal, 4PL nonlinear fit analysis in Prism software version 9 (GraphPad).

### Hybridoma generation.

A survivor of natural infection was identified and described previously (5), and a sample was collected a few months after negative PCR test. After written informed consent was obtained, peripheral blood was collected and stored at room temperature until PBMCs could be purified using SepMate tubes (STEMCELL Technologies) per the manufacturer’s protocol and then cryopreserved in 10% (v/v) dimethyl sulfoxide in FBS and stored in the vapor phase of liquid nitrogen. Sample derived in Spain were transferred to the Vanderbilt site. Approximately 10^7^ cryopreserved PBMCs were thawed, and lymphoblastoid cell lines (LCLs) were generated as previously described (45, 47) from memory B cells within the PBMCs by transformation with EBV (obtained from B95.8 cells) and supplemented with cell cycle checkpoint kinase 2 inhibitor (Sigma-Aldrich), CpG (Sigma-Aldrich), and cyclosporin A (Sigma-Aldrich) in Medium A (STEMCELL Technologies). One week later, LCLs were counted and then expanded on a feeder layer of γ-irradiated, human PBMCs from discarded leukofiltration devices. After 7 days, LCL supernatants were screened for the presence of CCHFV Gc-, Gn-, or GP38-recognizing antibodies using IbAr10200 strain M segment–transfected Expi293F cells on the Intellicyt iQue high-throughput flow cytometer as described below. LCLs from wells containing antibodies that bound to M segment–transfected cells were fused to HMMA2.5 myeloma cells by an established electrofusion technique (47). After fusion, hybridoma lines were cultured in a selection medium (with hypoxanthine, aminopterin, and thymidine medium supplements [Sigma-Aldrich] and ouabain [Sigma-Aldrich]) in 384-well cell culture plates before screening for CCHFV-specific antibody production in supernatants. Two weeks later, supernatants from the hybridoma cell lines were screened by binding and then cloned by single-cell flow cytometry sorting on a BD FACSAria III sorting cytometer (BD Biosciences) with aerosol containment in 384-well plates. These cloned cells were expanded in Medium E in 12-well tissue culture–treated plates (Corning) upon reaching 50% confluence, and their supernatants were screened for neutralizing activity. mAb-producing hybridoma cell lines were selected from wells displaying binding activity.

### Antibody production and purification.

For hybridoma-derived mAb, clonal cells were grown in 75 cm^2^ flasks to 70% confluency in hybridoma growth medium (ClonaCell-HY medium E from STEMCELL Technologies, 03805). The hybridoma cells were grown to exhaustion in Hybridoma-SFM (1×) serum-free medium (Gibco Hybridoma-SFM, Invitrogen, 12045084) in four 225 cm^2^ flasks. Exhausted hybridoma supernatant was harvested after 1 month. For the recombinant mAb production, the genes of heavy and light chains were synthesized into cDNA. The fragments were cloned into a full-length IgG1 DNA plasmid expression vector (48). The heavy and light chains were transformed into *Escherichia coli* cells to produce large amounts of DNA. Following the manufacturer’s protocol (Thermo Fisher Scientific), plasmids encoding heavy and light antibody chains were transiently transfected into Expi293F cells to produce mAb proteins. Secreted IgGs from recombinant and hybridomas were purified from filtered supernatants by affinity chromatography using Protein G columns (Cytiva, HiTrap Protein G HP columns) on an ÄKTA pure instrument. Purified mAbs were processed by buffer exchanging into PBS using Zeba Spin Desalting Columns (Thermo Fisher Scientific), filtering using sterile 0.45 μm Millipore filter devices, concentrated using Amicon Ultra-4 50 kDa centrifugal filter units (MilliporeSigma), and stored at –80°C. Recombinant mAbs were used for all in vivo experiments, and designated mAbs were either hybridoma or recombinant derived in in vitro experiments.

### mAb isotype and gene sequencing analysis.

The antibody heavy- and light-chain variable region genes were obtained from hybridoma cell lines that had been cloned by flow cytometry single-cell sorting. Total RNA extraction was performed using the RNeasy Mini Kit (Qiagen) per the manufacturer’s protocol. Amplification of cDNA ends was done using a modified 5′ rapid amplification approach (49). Briefly, a mixture of 5 μL of total RNA and cDNA synthesis primer mix (10 μM each) was incubated at 70°C (2 minutes), followed by a 42°C incubation step (1–3 minutes) for synthesis primer annealing. After incubation, a mixture of 5× first-strand buffer (Clontech), dithiothreitol (20 mM), 5′ template switch oligo (10 μM), deoxynucleotide triphosphate (dNTP) solution (10 mM each), and 10× SMARTScribe Reverse Transcriptase (Clontech) (60-minute incubation) was added to the total RNA reaction at 42°C. The first-strand synthesis reaction was purified using the AMPure Size Select Magnetic Bead Kit (ratio of 1.8×; Beckman Coulter). After purification, a single PCR amplification reaction with 5 μL of first-strand cDNA, 2× Q5 High-Fidelity Master Mix (New England Biolabs), dNTP (10 mM each), forward universal primer (10 μM), and reverse primer mix (0.2 μM each in heavy-chain mix and 0.2 μM each in light-chain mix) was subjected to the following: initial denaturation for 90 seconds followed by 30 cycles of denaturation at 98°C for 10 seconds, annealing at 60°C for 20 seconds, and extension at 72°C for 40 seconds, followed by a final extension step at 72°C for 4 minutes. Primers used here were previously detailed (48, 49). Using the AMPure Size Select Magnetic Bead Kit (ratio of 0.6×), the first PCR reaction was purified. Amplicon libraries were prepared per the Multiplex SMRT Sequencing protocol (Pacific Biosciences) and sequenced on a Sequel instrument (Pacific Biosciences). Raw sequences were demultiplexed, and circular consensus sequences were determined using SMRT Analysis tool suite (Pacific Biosciences). The identities of gene segments, complementarity-determining regions, and mutations were determined using the ImMunoGeneTics database (50).

### mAb competition-binding assay using a Gc-Gn cell display system.

For antibody preparation, mAbs were directly fluorescently labeled. Briefly, mAbs were labeled with Alexa Fluor 647 NHS ester (Thermo Fisher Scientific) following the manufacturer’s protocol. Labeled mAbs were purified and buffer exchanged into PBS using desalting Zeba columns (Thermo Fisher Scientific) and stored at 4°C until use. For cell display of Gc-Gn, plasmid encoding full-length M segment of CCHFV IbAr10200 was transiently transfected into Expi293F cells per the manufacturer’s protocol (Thermo Fisher Scientific). Cells were cultured to produce antigen for 2 days, and cells were either processed for staining or frozen and stored in the vapor phase of liquid nitrogen until use only if cells were > 80% viable. Cells (1 × 10^7^) were fixed and permeabilized using BD Cytofix Cytoperm according to the manufacturer’s protocol. Cells (50,000/well) then were plated into 96-well V-bottom plates in permeabilization buffer. Cells were incubated with a saturating concentration (typically 20 μg/mL) of the first unlabeled mAb at room temperature for 30 minutes. The second fluorescently labeled mAb (5 μg/mL) was added for 30 minutes without prewashing to minimize the dissociation of the first mAb. Cells were washed, fixed in 1% PFA, and resuspended in 30 μL of FACS buffer (DPBS, 2 mM EDTA, and 2% FBS). Staining was analyzed using an iQue flow cytometer, and MFI values were used for analysis. Background values were determined from binding of second-labeled mAb to mock-transfected Expi293F cells. Results are expressed as the percent of binding in the presence of competitor mAb minus background over second mAb only (maximal binding) minus background. Antibodies were considered competing if the presence of the first antibody reduced the signal of the second antibody to < 30% of its maximal binding or noncompeting if the signal was > 70%. Similar assay protocol and outlay were performed to compete the mouse antibodies with human antibodies using the preGn construct, except that mouse or human antibodies were used as the primary antibodies (at 20 μg/mL), as indicated in Figure 7.

### mAb binding assay using a Gc-Gn cell display system.

mAbs were serially diluted in PBS and added to fixed and permeabilized (BD Biosciences) CCHFV M segment–transfected 293F cells (GPC of CCHFV isolates: ArD15786 [DQ211627], Baghdad-12 [AJ538197], Kosova Hoti [EU037902], NIV112143 [JN572085], SPU18/88 [KJ682810], Sudan Al-Fulah 3–2008 [HQ378185], YL04057 [FJ562094], IbAr10200, Oman199809166, Turkey200406546, and Afg09) (25). CCHFV M segment–transfected 293F cells were fixed and permeabilized using a BD Biosciences kit, per manufacturer’s protocol, and added to V-bottom 96-well plates. A Gc construct for transient expression was designed to express Gc (amino acids 1041–1684) under the MKVIWFSSLICFVIQCSG signal sequence. The preGn-expressing plasmids were also under the same signal sequence while expressing amino acids 23–842 with the RSKR cleavage site altered to RRLL to allow for proper cleavage without cotrafficking along with the Gc glycoprotein. Both constructs were independently transfected similarly to the full-length constructs and designed from the sequence deposited online with genome ID 1980519.4147 at the Bacterial and Viral Bioinformatics Resource Center (https://www.bv-brc.org/). To detect antibody binding to Gc, preGn, or full-length GPC, mAbs were serially diluted in permeabilization buffer and added to prepared cells expressing their respective constructs for 30 minutes at room temperature. After incubation, cells were washed with permeabilization buffer and secondary antibody (anti-human PE-conjugated, Southern Biotech, 1:500 dilution, catalog 2040-09) was added in permeabilization buffer for 30 minutes at room temperature. Cells were washed in FACS buffer (PBS, 10 mM EDTA, and 2% FBS) and assessed using an iQue flow cytometer. Untransfected cells were used as the negative control.

### CCHFV challenge in mice.

In the prophylaxis study, experimental groups (*n* = 8; 5 female and 3 male) and the control group (*n* = 4; all female) consisted of adult STAT1-KO BL/6 mice from Taconic Biosciences. Animals were given 1 mg of CCHF-82 IgG1 or control doses of vehicle or isotype control 1 day before challenge via the i.p. route. Animals were challenged with 100 PFUs of CCHFV Turkey 200406546 strain via the i.p. route. Animals were monitored for weight loss, temperature, and mortality with clinical scoring through day 28 after challenge. In the treatment study, experimental groups (*n* = 6 for the treatment group; 3 male and 3 female in each group) in the postexposure study consisted of adult STAT1-KO BL/6 mice from Taconic Biosciences. Animals were inoculated with 100 PFUs of CCHFV Turkey 200406546 strain by the i.p. route. Animals were treated with antibody (250 μg for all antibodies, except CCHF-135 at 125 μg) once i.p. at 30 minutes after infection. Human mAb DENV-2D22 (specific to an unrelated target, dengue virus) was used as the negative control. Mice were monitored daily from 0 to 27 days after infection for survival and body weight, and survivors were euthanized 27 days after infection. Clinical scores indicate the following: 1, healthy; 2, ruffled fur and/or lethargic; 3, a score of 2 plus 1 additional clinical sign, such as hunched posture, orbital tightening, and/or >10% weight loss; 4, a score of 3 plus 1 additional clinical sign, such as reluctance to move when stimulated and/or >15% weight loss; and 5, >25% weight loss for >2 days and >25% weight loss with clinical signs scoring 2–4, inability to reach food /water normally, and any neurologic signs. If animals were found moribund, they were immediately euthanized. Prophylactic studies were performed with recombinant IgG1 forms of the antibody, while postexposure prophylactic studies were performed with hybridoma-derived lots of the antibodies.

### Gn ELISA.

For ELISA binding assessment of mAbs, 384-well ELISA plates were directly coated with Gn recombinant protein (The Native Antigen Company, REC31615) at 2 μg/mL (diluted in PBS) and incubated overnight at 4°C. Plates were washed 3 times with PBST using an EL406 combination washer dispenser instrument (BioTek) and blocked for 1 hour at room temperature with 5% milk powder and 2% goat serum (diluted in PBS). After washing 3 times with PBST, 30 μL serum diluted in PBS was added to plates and incubated for 1 hour at room temperature. Plates were then washed 3 times, and 30 μL of goat anti-human HRP-conjugated secondary antibodies (SouthernBiotech, catalog 2047-05) diluted 1:1,000 in PBS was added to plates and incubated for 1 hour at room temperature. Plates were washed 3 times, and 25 μL of 1-Step Ultra TMB-ELISA (Thermo Fisher Scientific, 34029) was added for 7 minutes, and the reaction was quenched with 25 μL of 1 M HCl. Plates were read for optical density at 450 nm immediately using a BioTek plate reader.

### Statistics.

Kaplan-Meier survival curves were analyzed using the Mantel-Cox log-rank test. A *P* value < 0.05 was considered significant. Differences between groups were analyzed by Fisher’s exact (2-tailed) test. Technical and biological replicates are indicated in Methods and figure legends. Data are presented as the mean calculated from replicate samples and SD between replicates. Statistical analyses were performed using Prism version 9 (GraphPad).

### Study approval.

Human PBMCs were collected from a survivor of natural infection following informed written consent through a program maintained by the Instituto de Salud Carlos III. This project was approved by the ethics and research committee of the Instituto de Salud Carlos III. Serum samples were obtained in Turkey, following informed written consent from convalescent patients. These samples were acquired under the approval of the ethics and research committee of Maramara University. The studies also were approved by the Vanderbilt University Medical Center Institutional Review Board. Animal studies and live-virus assays were performed at the Galveston National Laboratory, UTMB. These studies were approved by the UTMB IACUC. The studies were approved by the UTMB Institutional Biosafety Committees and conducted in Select Agent–approved animal BSL-4 facilities that are fully accredited by the Association for Assessment and Accreditation of Laboratory Animal Care International.

### Data availability.

Antibody sequences are available in GenBank with accession numbers PX975503–PX975568 (light chains) and PX975569–PX975634 (heavy chains). Further information and requests for resources and reagents should be directed to and will be fulfilled by the corresponding author. Materials described in this paper are available for distribution for nonprofit use using the MTA Toolkit from the Association of University Technology Managers available at https://autm.net/surveys-and-tools/agreements/material-transfer-agreements/mta-toolkit All relevant and source data for the main text figures are available in the Supporting Data Values file.

## Author contributions

NSC, RWC, TWG, and JEC conceived the project. JEC and TWG obtained funding. NSC, RWC, VB, NK, and SP performed laboratory experiments. EB provided critical reagents and advice. ETE, MPSS, and JM obtained human blood specimens. NSC, LM, and JEC analyzed data. TWG and JEC supervised the research. NSC and JEC wrote the first draft of the paper. All authors reviewed and approved the final manuscript.

## Conflict of interest

JEC has served as a consultant for Luna Innovations, Merck Sharp & Dohme, Emergent Biosolutions, and GlaxoSmithKline and is a member of the Scientific Advisory Board of Meissa Vaccines, a former member of the Scientific Advisory Board of Gigagen (Grifols), and founder of IDBiologics. The laboratory of JEC received unrelated sponsored research agreements from AstraZeneca, Takeda, and IDBiologics during the study. Vanderbilt University has applied for patents for some of the antibodies in this paper.

## Funding support

This work is the result of NIH funding, in whole or in part, and is subject to the NIH Public Access Policy. Through acceptance of this federal funding, the NIH has been given a right to make the work publicly available in PubMed Central.

- NIH grant R01AI132246 (to TWG) and UC7AI094660 for BSL-4 operations support of the Galveston National Laboratory.
- National Institute of Allergy and Infectious Diseases of the NIH award T32 AI007151 (to NSC).

## Supplementary Material

Supplemental data

Supporting data values

## Version 1 03/31/2026

In-Press Preview

## Version 2 05/15/2026

Electronic publication
