# Supplementary material for: Human antibody targeting Crimean-Congo hemorrhagic fever virus glycoprotein 38 protects mice against heterologous virus challenge
Source: J Clin Invest. 2026 Mar 31;136(10):e191440. doi: 10.1172/JCI191440 (PMC13178664; doi:10.1172/JCI191440)
Supplement: Supplemental data [file jci-136-191440-s158.pdf]

Supplemental Figures for

Human antibody targeting Crimean-Congo hemorrhagic fever virus glycoprotein 38 protects mice against heterologous virus challenge

Nathaniel S. Chapman[1,2], Viktoriya Borisevich[3], Nurgun Kose[2], Luke Myers[2], Stephen G. Priest[2], Éric Bergeron[4,5], Elena Trigo-Esteban[6], María Paz Sánchez-Seco Fariñas[7], José Antonio Melero Fondevila[7,8,*], Thomas W. Geisbert[3,9,10], Robert W. Cross[3,9,10], James E. Crowe, Jr.[1,2,11]

This file contains:

Figures S1-S8

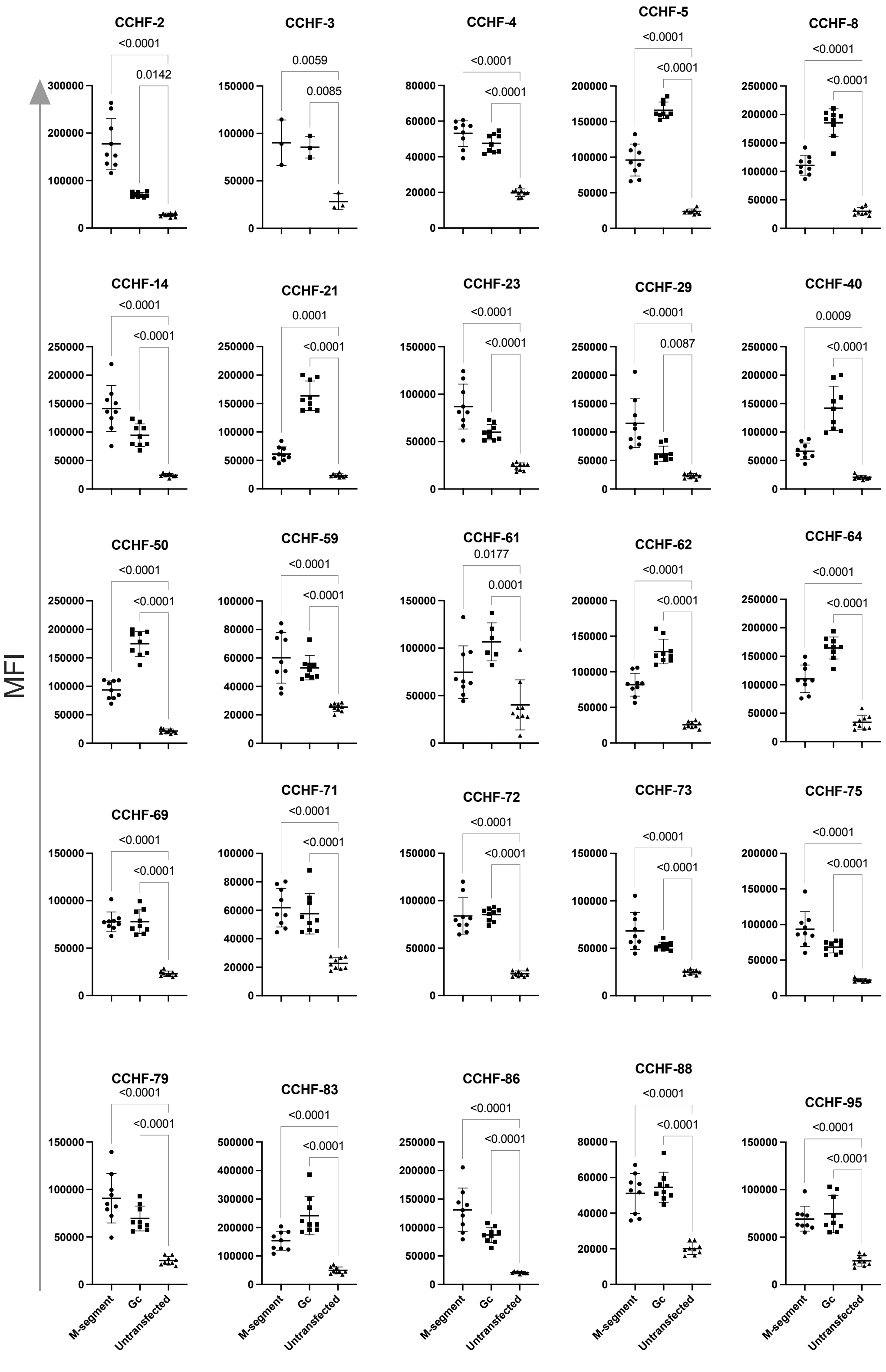
MFI
CCHF-2
<0.0001
0.0142
CCHF-3
0.0059
0.0085
CCHF-4
<0.0001
<0.0001
CCHF-5
<0.0001
<0.0001
CCHF-8
<0.0001
<0.0001
CCHF-14
<0.0001
<0.0001
CCHF-21
0.0001
<0.0001
CCHF-23
<0.0001
<0.0001
CCHF-29
<0.0001
0.0087
CCHF-40
0.0009
<0.0001
CCHF-50
<0.0001
<0.0001
CCHF-59
<0.0001
<0.0001
CCHF-61
0.0177
0.0001
CCHF-62
<0.0001
<0.0001
CCHF-64
<0.0001
<0.0001
CCHF-69
<0.0001
<0.0001
CCHF-71
<0.0001
<0.0001
CCHF-72
<0.0001
<0.0001
CCHF-73
<0.0001
<0.0001
CCHF-75
<0.0001
<0.0001
CCHF-79
<0.0001
<0.0001
CCHF-83
<0.0001
<0.0001
CCHF-86
<0.0001
<0.0001
CCHF-88
<0.0001
<0.0001
CCHF-95
<0.0001
<0.0001
M-segment
Gc
Untransfected

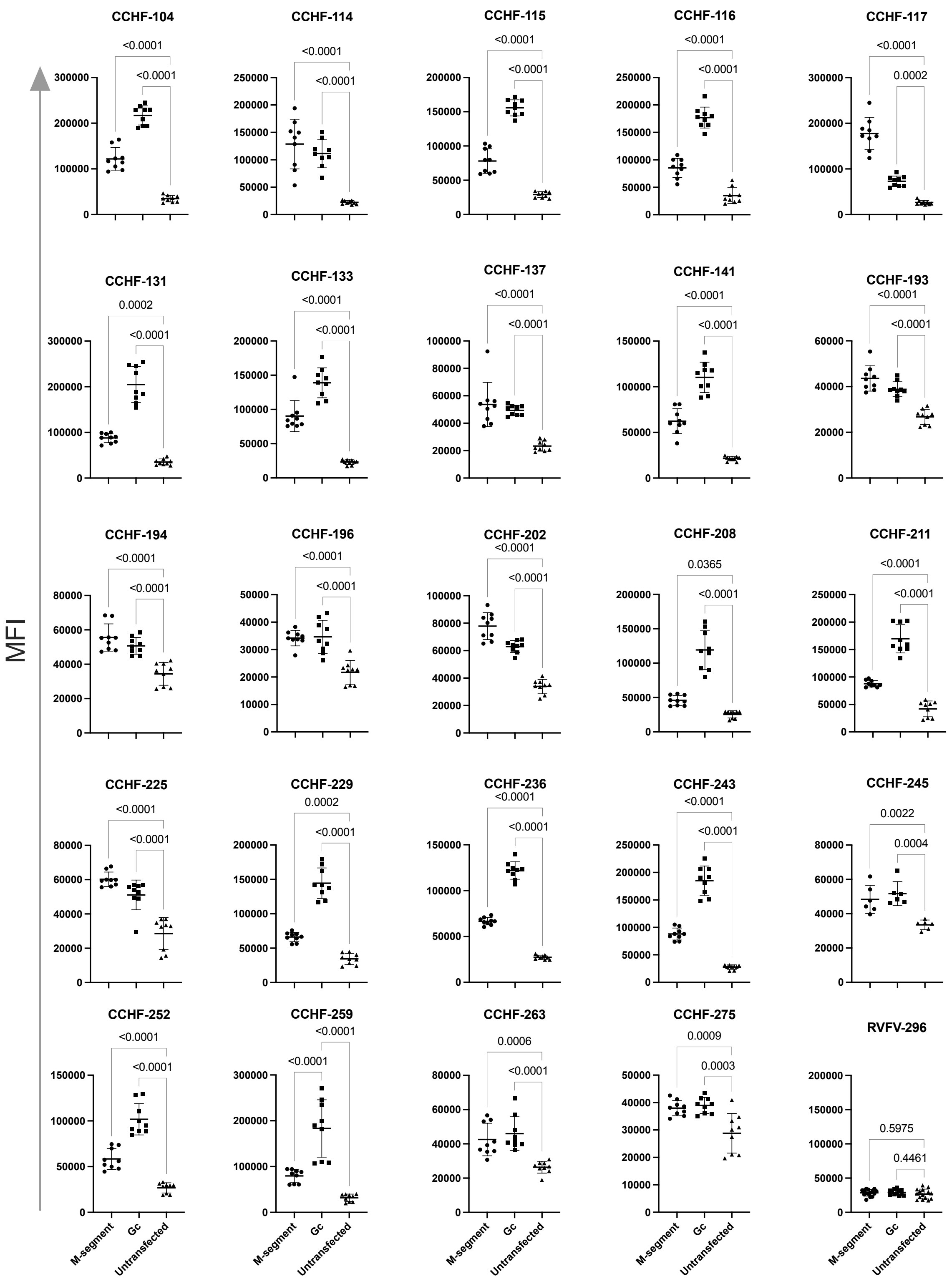
MFI
CCHF-104
<0.0001
<0.0001
CCHF-114
<0.0001
<0.0001
CCHF-115
<0.0001
<0.0001
CCHF-116
<0.0001
<0.0001
CCHF-117
<0.0001
0.0002
CCHF-131
0.0002
<0.0001
CCHF-133
<0.0001
<0.0001
CCHF-137
<0.0001
<0.0001
CCHF-141
<0.0001
<0.0001
CCHF-193
<0.0001
<0.0001
CCHF-194
<0.0001
<0.0001
CCHF-196
<0.0001
<0.0001
CCHF-202
<0.0001
<0.0001
CCHF-208
0.0365
<0.0001
CCHF-211
<0.0001
<0.0001
CCHF-225
<0.0001
<0.0001
CCHF-229
0.0002
<0.0001
CCHF-236
<0.0001
<0.0001
CCHF-243
<0.0001
<0.0001
CCHF-245
0.0022
0.0004
CCHF-252
<0.0001
<0.0001
CCHF-259
<0.0001
<0.0001
CCHF-263
0.0006
<0.0001
CCHF-275
0.0009
0.0003
RVFV-296
0.5975
0.4461
M-segment
Gc
Untransfected

Supplemental Figure 1. Subcomponent of the panel of human monoclonal antibodies that recognize the Gc glycoprotein. Human antibodies were incubated with cells transiently expressing either full length M-segment or Gc of the IbAr10200 strain of CCHFV and compared to untransfected cells. Data represents three independent experiments with three replicates each. Error bars represent mean +/- SD and *P* values are shown for each condition vs untransfected cells. Ordinary one-way ANOVA using Dunnett's multiple comparisons in Prism 9 software (Graphpad) were performed for statistical analysis.

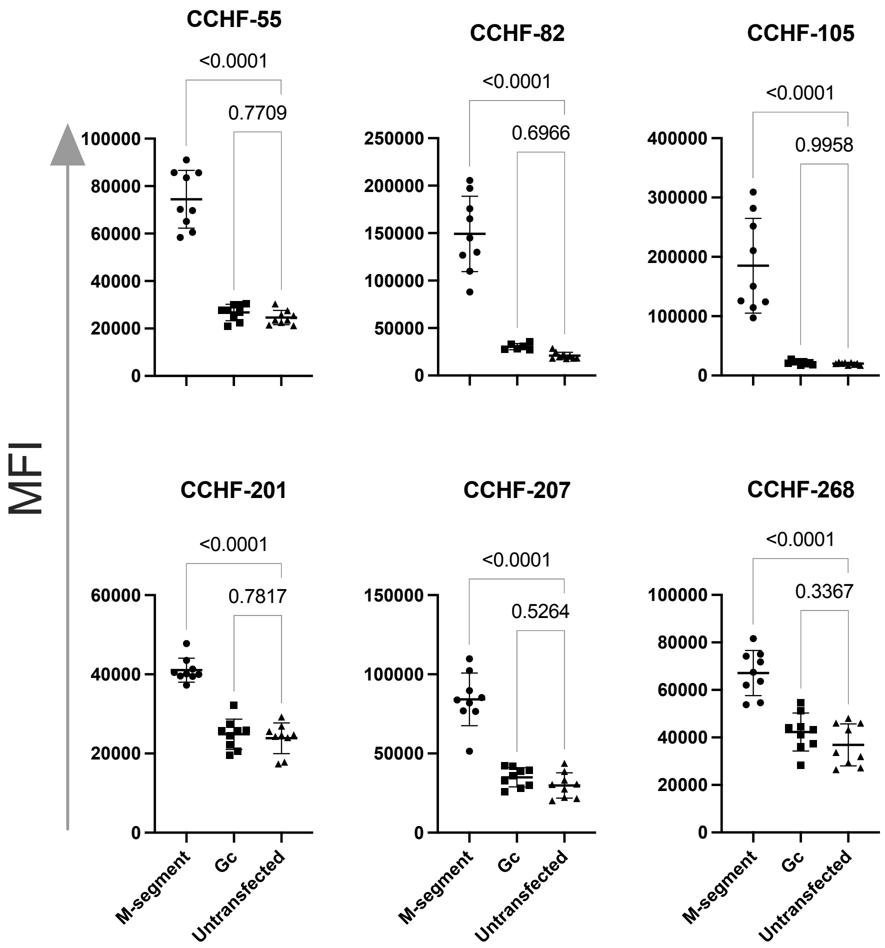

Supplemental Figure 2. Subcomponent of the panel of human monoclonal antibodies that recognize the preGn glycoprotein precursor. Human antibodies were incubated with cells transiently expressing either full length M-segment or Gc of the IbAr10200 strain of CCHFV and compared to untransfected cells. Data represents three independent experiments with three replicates each. Error bars represent mean +/- SD and *P* values are shown for each condition vs untransfected cells. Ordinary one-way ANOVA using Dunnett's multiple comparisons in Prism 9 software (Graphpad) were performed for statistical analysis.

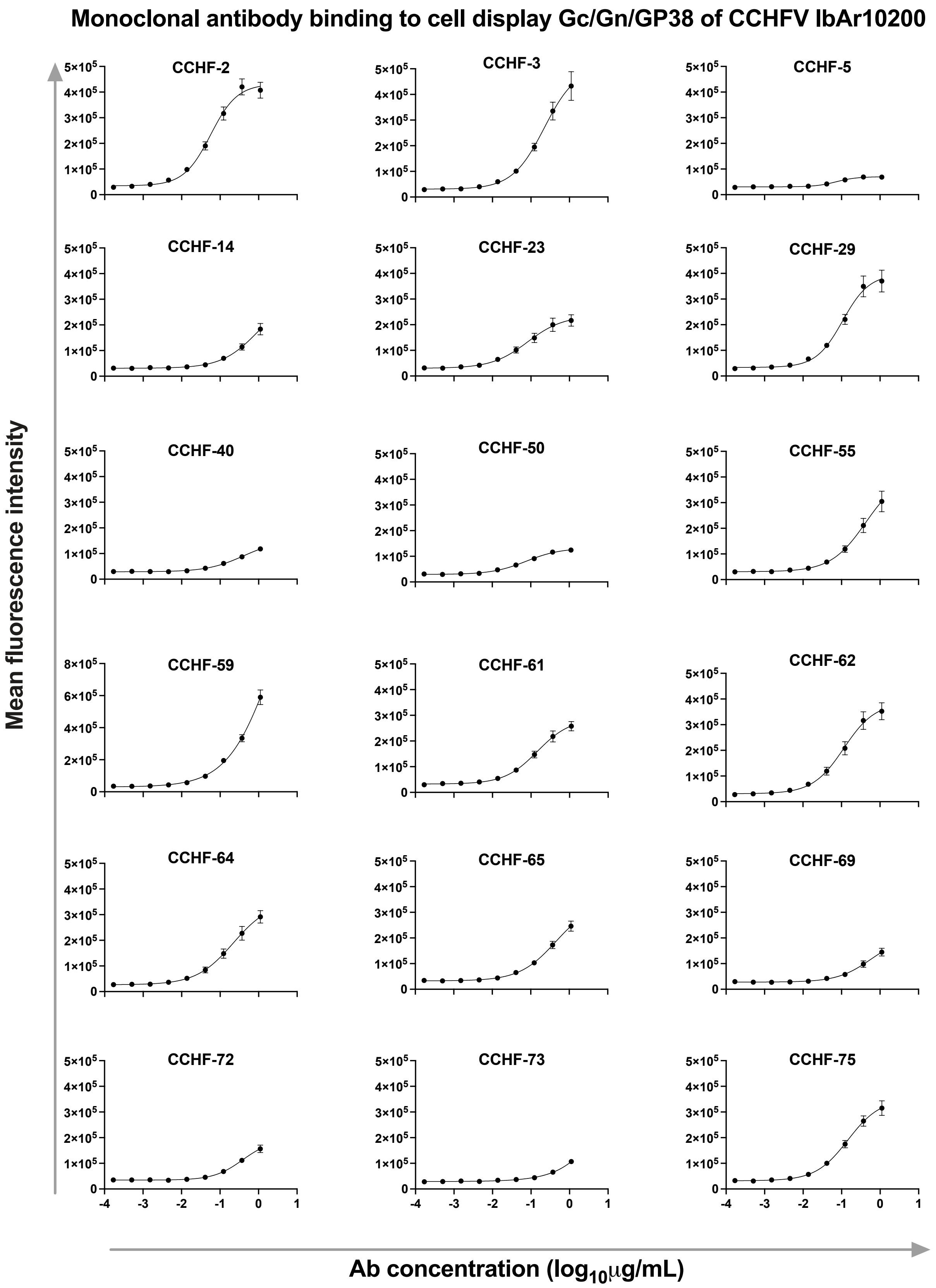
Monoclonal antibody binding to cell display Gc/Gn/GP38 of CCHFV IbAr10200
CCHF-2
CCHF-3
CCHF-5
CCHF-14
CCHF-23
CCHF-29
CCHF-40
CCHF-50
CCHF-55
CCHF-59
CCHF-61
CCHF-62
CCHF-64
CCHF-65
CCHF-69
CCHF-72
CCHF-73
CCHF-75
Mean fluorescence intensity
Ab concentration (log10μg/mL)

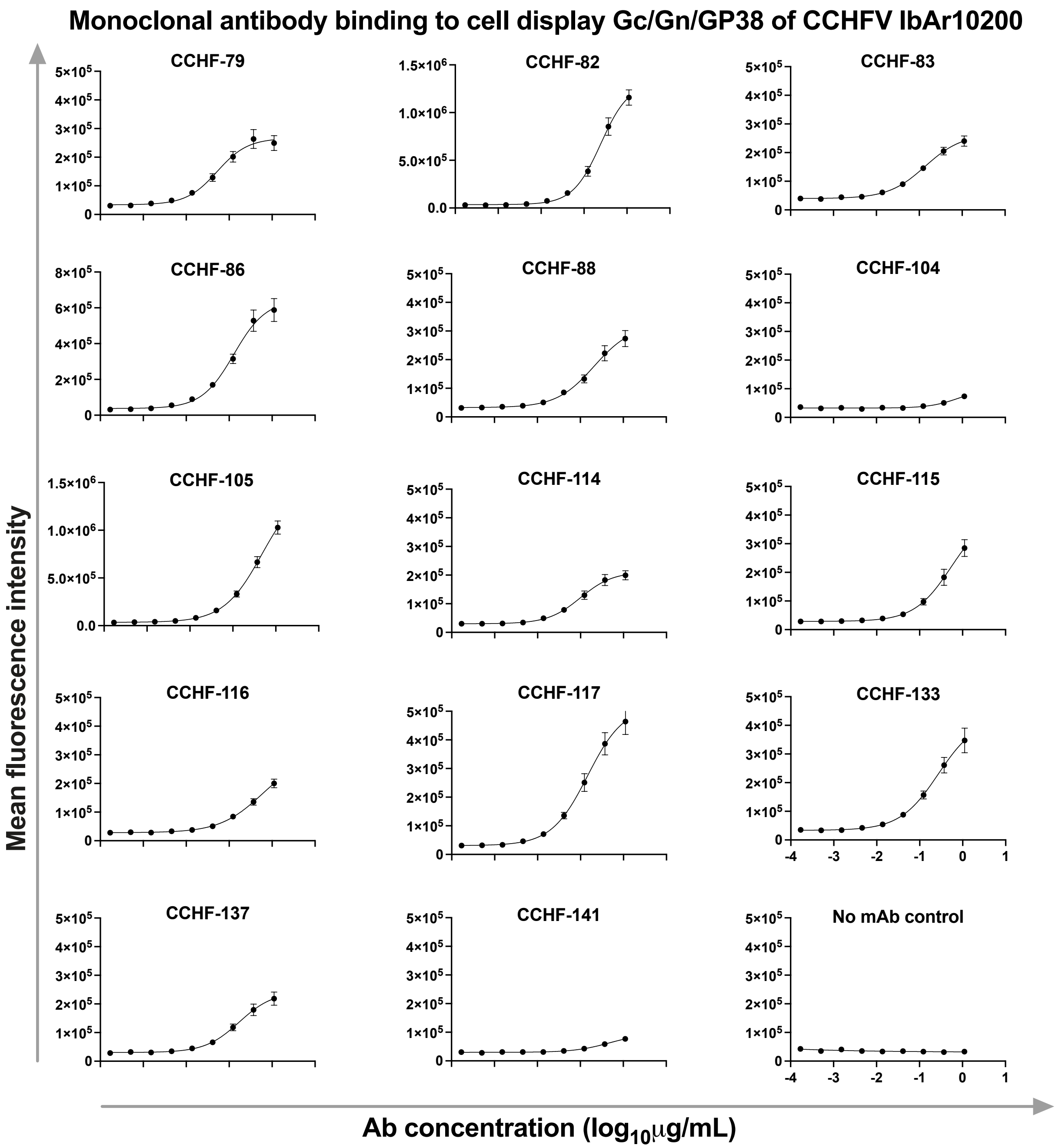

Supplemental Figure 3. Human antibodies derived from the hybridoma process from a survivor of CCHFV infection bind to M-segment transfected cells. A panel of human monoclonal antibodies were titrated on M-segment expressing cells. Assay was performed in biological triplicate and technical duplicates. Data were analyzed using a sigmoidal, 4PL nonlinear fit analysis in Prism software version 9 (GraphPad). Error bars represent mean +/- SEM.

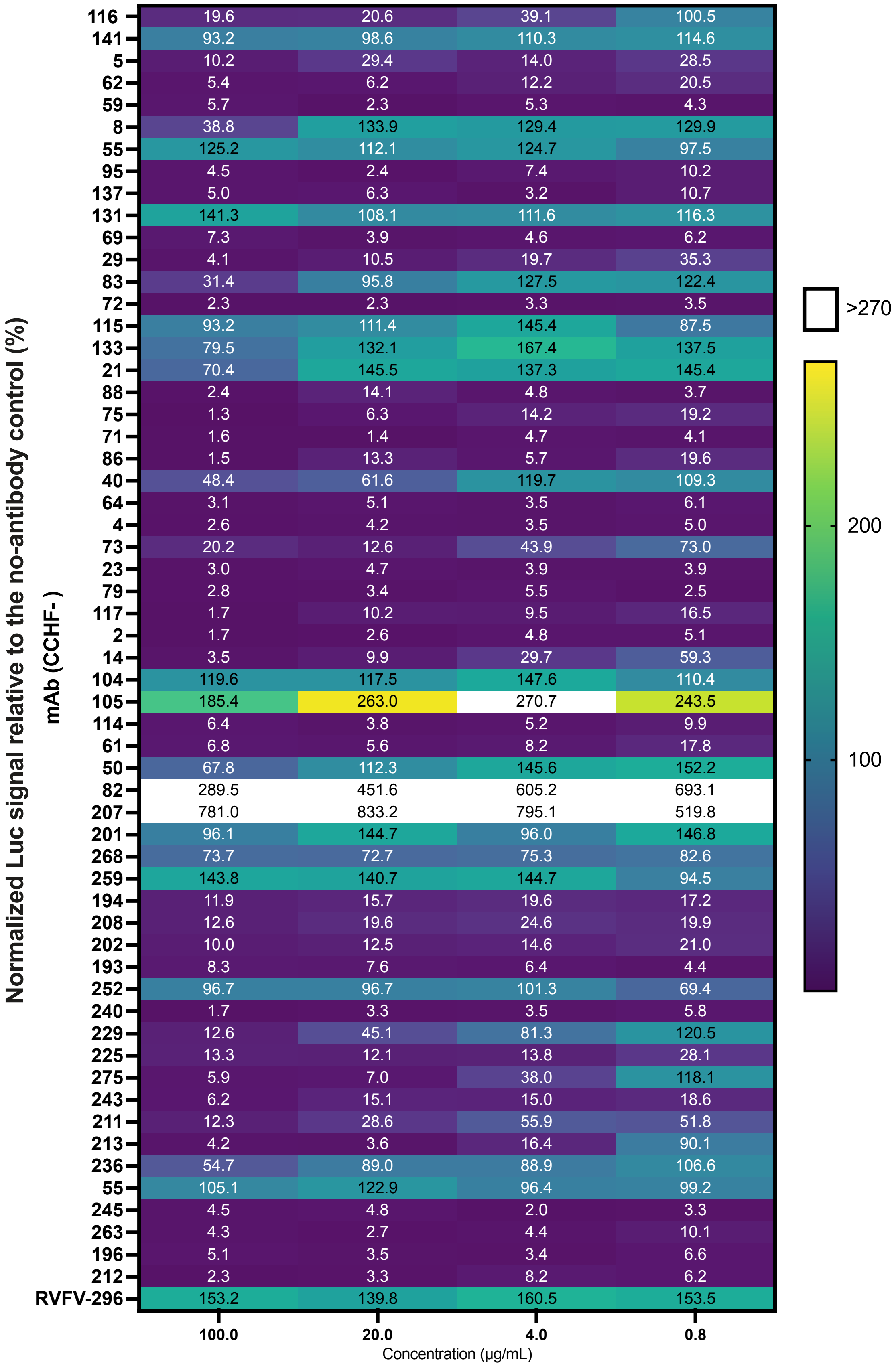
Normalized Luc signal relative to the no-antibody control (%)
mAb (CCHF-)
116 19.6 20.6 39.1 100.5
141 93.2 98.6 110.3 114.6
5 10.2 29.4 14.0 28.5
62 5.4 6.2 12.2 20.5
59 5.7 2.3 5.3 4.3
8 38.8 133.9 129.4 129.9
55 125.2 112.1 124.7 97.5
95 4.5 2.4 7.4 10.2
137 5.0 6.3 3.2 10.7
131 141.3 108.1 111.6 116.3
69 7.3 3.9 4.6 6.2
29 4.1 10.5 19.7 35.3
83 31.4 95.8 127.5 122.4
72 2.3 2.3 3.3 3.5
115 93.2 111.4 145.4 87.5
133 79.5 132.1 167.4 137.5
21 70.4 145.5 137.3 145.4
88 2.4 14.1 4.8 3.7
75 1.3 6.3 14.2 19.2
71 1.6 1.4 4.7 4.1
86 1.5 13.3 5.7 19.6
40 48.4 61.6 119.7 109.3
64 3.1 5.1 3.5 6.1
4 2.6 4.2 3.5 5.0
73 20.2 12.6 43.9 73.0
23 3.0 4.7 3.9 3.9
79 2.8 3.4 5.5 2.5
117 1.7 10.2 9.5 16.5
2 1.7 2.6 4.8 5.1
14 3.5 9.9 29.7 59.3
104 119.6 117.5 147.6 110.4
105 185.4 263.0 270.7 243.5
114 6.4 3.8 5.2 9.9
61 6.8 5.6 8.2 17.8
50 67.8 112.3 145.6 152.2
82 289.5 451.6 605.2 693.1
207 781.0 833.2 795.1 519.8
201 96.1 144.7 96.0 146.8
268 73.7 72.7 75.3 82.6
259 143.8 140.7 144.7 94.5
194 11.9 15.7 19.6 17.2
208 12.6 19.6 24.6 19.9
202 10.0 12.5 14.6 21.0
193 8.3 7.6 6.4 4.4
252 96.7 96.7 101.3 69.4
240 1.7 3.3 3.5 5.8
229 12.6 45.1 81.3 120.5
225 13.3 12.1 13.8 28.1
275 5.9 7.0 38.0 118.1
243 6.2 15.1 15.0 18.6
211 12.3 28.6 55.9 51.8
213 4.2 3.6 16.4 90.1
236 54.7 89.0 88.9 106.6
55 105.1 122.9 96.4 99.2
245 4.5 4.8 2.0 3.3
263 4.3 2.7 4.4 10.1
196 5.1 3.5 3.4 6.6
212 2.3 3.3 8.2 6.2
RVFV-296 153.2 139.8 160.5 153.5
100.0
20.0
4.0
0.8
Concentration (µg/mL)
>270
200
100

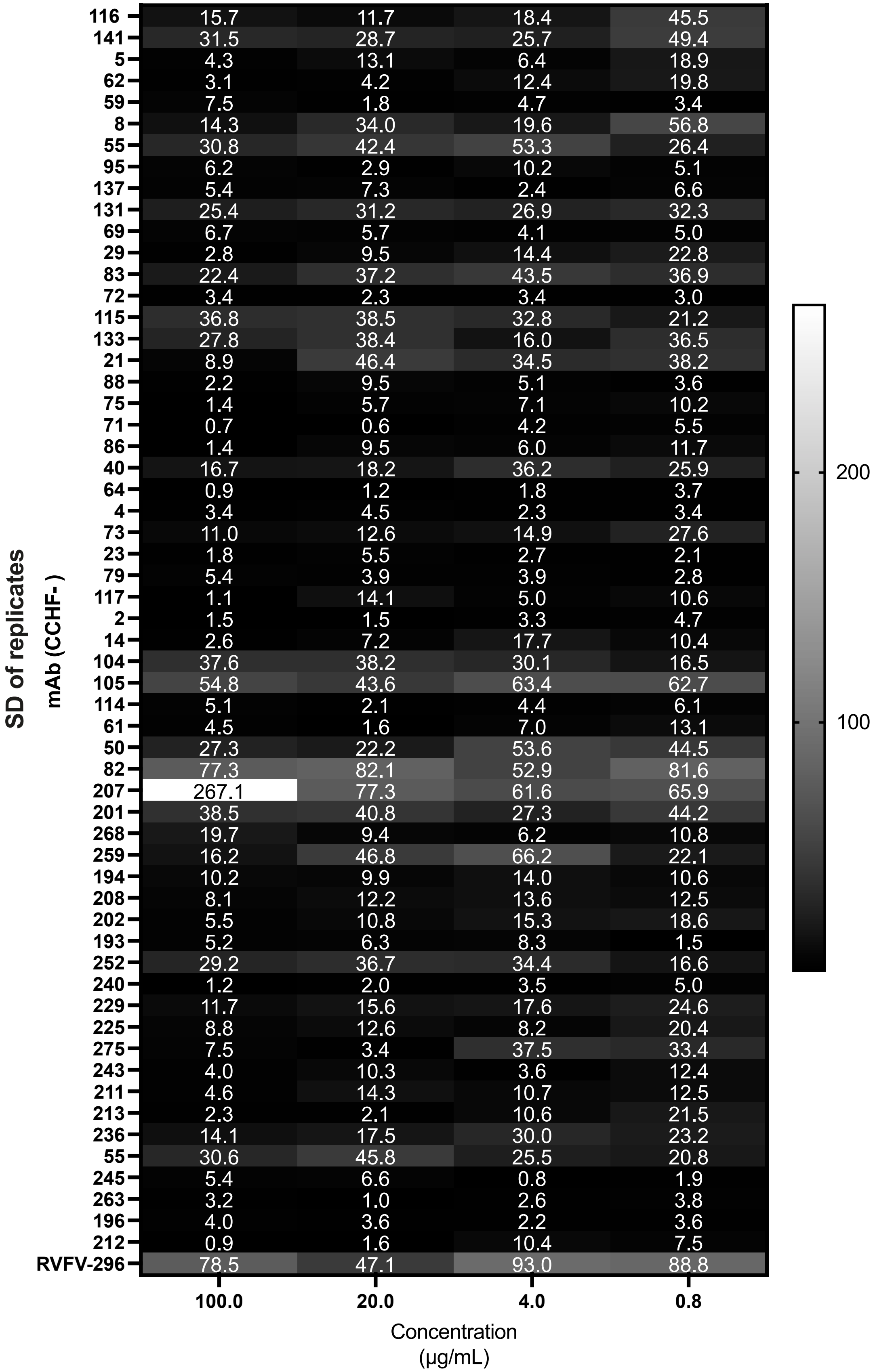
SD of replicates
mAb (CCHF-)
116 15.7 11.7 18.4 45.5
141 31.5 28.7 25.7 49.4
5 4.3 13.1 6.4 18.9
62 3.1 4.2 12.4 19.8
59 7.5 1.8 4.7 3.4
8 14.3 34.0 19.6 56.8
55 30.8 42.4 53.3 26.4
95 6.2 2.9 10.2 5.1
137 5.4 7.3 2.4 6.6
131 25.4 31.2 26.9 32.3
69 6.7 5.7 4.1 5.0
29 2.8 9.5 14.4 22.8
83 22.4 37.2 43.5 36.9
72 3.4 2.3 3.4 3.0
115 36.8 38.5 32.8 21.2
133 27.8 38.4 16.0 36.5
21 8.9 46.4 34.5 38.2
88 2.2 9.5 5.1 3.6
75 1.4 5.7 7.1 10.2
71 0.7 0.6 4.2 5.5
86 1.4 9.5 6.0 11.7
40 16.7 18.2 36.2 25.9
64 0.9 1.2 1.8 3.7
4 3.4 4.5 2.3 3.4
73 11.0 12.6 14.9 27.6
23 1.8 5.5 2.7 2.1
79 5.4 3.9 3.9 2.8
117 1.1 14.1 5.0 10.6
2 1.5 1.5 3.3 4.7
14 2.6 7.2 17.7 10.4
104 37.6 38.2 30.1 16.5
105 54.8 43.6 63.4 62.7
114 5.1 2.1 4.4 6.1
61 4.5 1.6 7.0 13.1
50 27.3 22.2 53.6 44.5
82 77.3 82.1 52.9 81.6
207 267.1 77.3 61.6 65.9
201 38.5 40.8 27.3 44.2
268 19.7 9.4 6.2 10.8
259 16.2 46.8 66.2 22.1
194 10.2 9.9 14.0 10.6
208 8.1 12.2 13.6 12.5
202 5.5 10.8 15.3 18.6
193 5.2 6.3 8.3 1.5
252 29.2 36.7 34.4 16.6
240 1.2 2.0 3.5 5.0
229 11.7 15.6 17.6 24.6
225 8.8 12.6 8.2 20.4
275 7.5 3.4 37.5 33.4
243 4.0 10.3 3.6 12.4
211 4.6 14.3 10.7 12.5
213 2.3 2.1 10.6 21.5
236 14.1 17.5 30.0 23.2
55 30.6 45.8 25.5 20.8
245 5.4 6.6 0.8 1.9
263 3.2 1.0 2.6 3.8
196 4.0 3.6 2.2 3.6
212 0.9 1.6 10.4 7.5
RVFV-296 78.5 47.1 93.0 88.8
100.0 20.0 4.0 0.8
Concentration
(µg/mL)
200
100

Supplemental Figure 4. Four-point concentration neutralization using the CCHFV panel isolated herein with the CCHFV tecVLP encoding the IbAr10200 GPC. A) CCHFV-specific mAbs were diluted to 100, 20, 4, and 0.8 μg/mL with tecVLP. The mixture was added to BHK-21 T7 cells and processed as described in methodology. RVFV-296 was used as the negative control. Percent infection is graded on a color scale as represented to the side of the table with the white boxes representing >270% infection relative to no antibody control. Data represent mean values. Assay was performed in biological triplicate and technical duplicates. **B**) Heatmap representing the SD of each antibody and concentration in concordance with Supplemental Figure 4A. The black and white scale table represents the SD values to enhance visual representation.

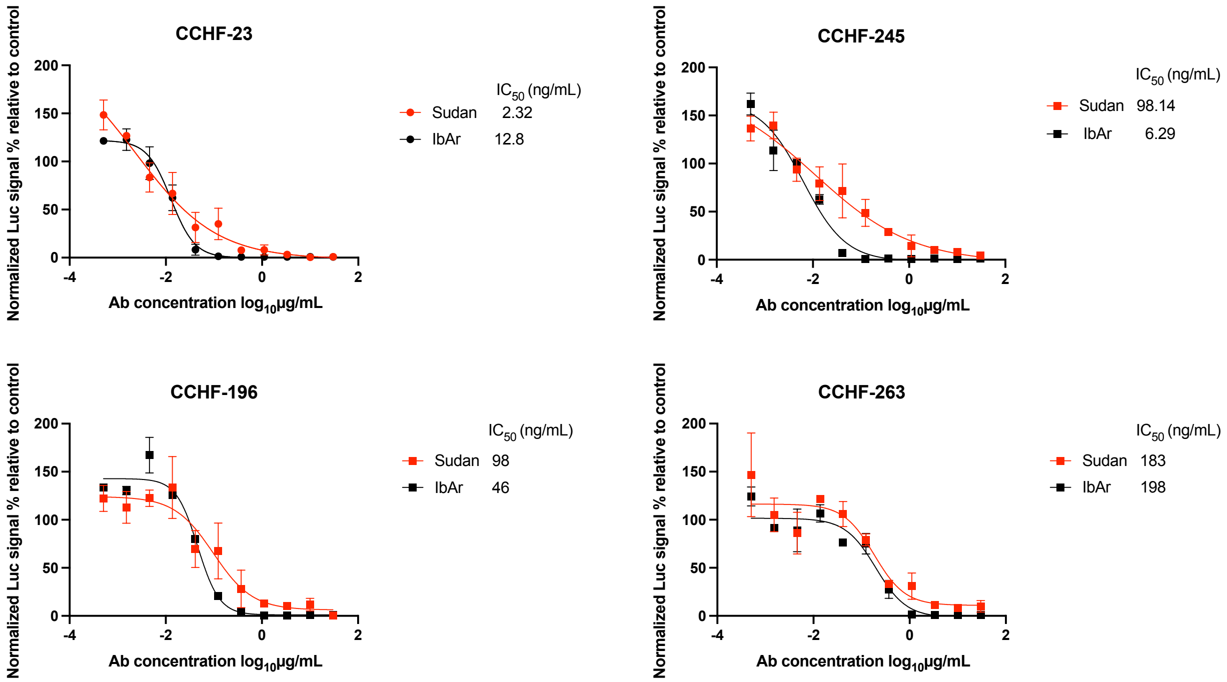

Supplemental Figure 5. Further titration of most neutralizing antibodies against CCHFV identified in our panel against tecVLP strains Sudan and IbAr10200.

The most potently neutralizing antibodies were serially titrated and mixed with 5000 luciferase units of tecVLP from with GPC expressing the Sudan or IbAr10200 strain before being added to BHK21-T7 cells and allowed to incubate for 24 hours. The assay was performed once with technical duplicate values. Values were normalized relative to no antibody control. Data were analyzed using a sigmoidal, 4PL nonlinear fit analysis in Prism software version 9 (GraphPad). Data represents mean +/- SEM.

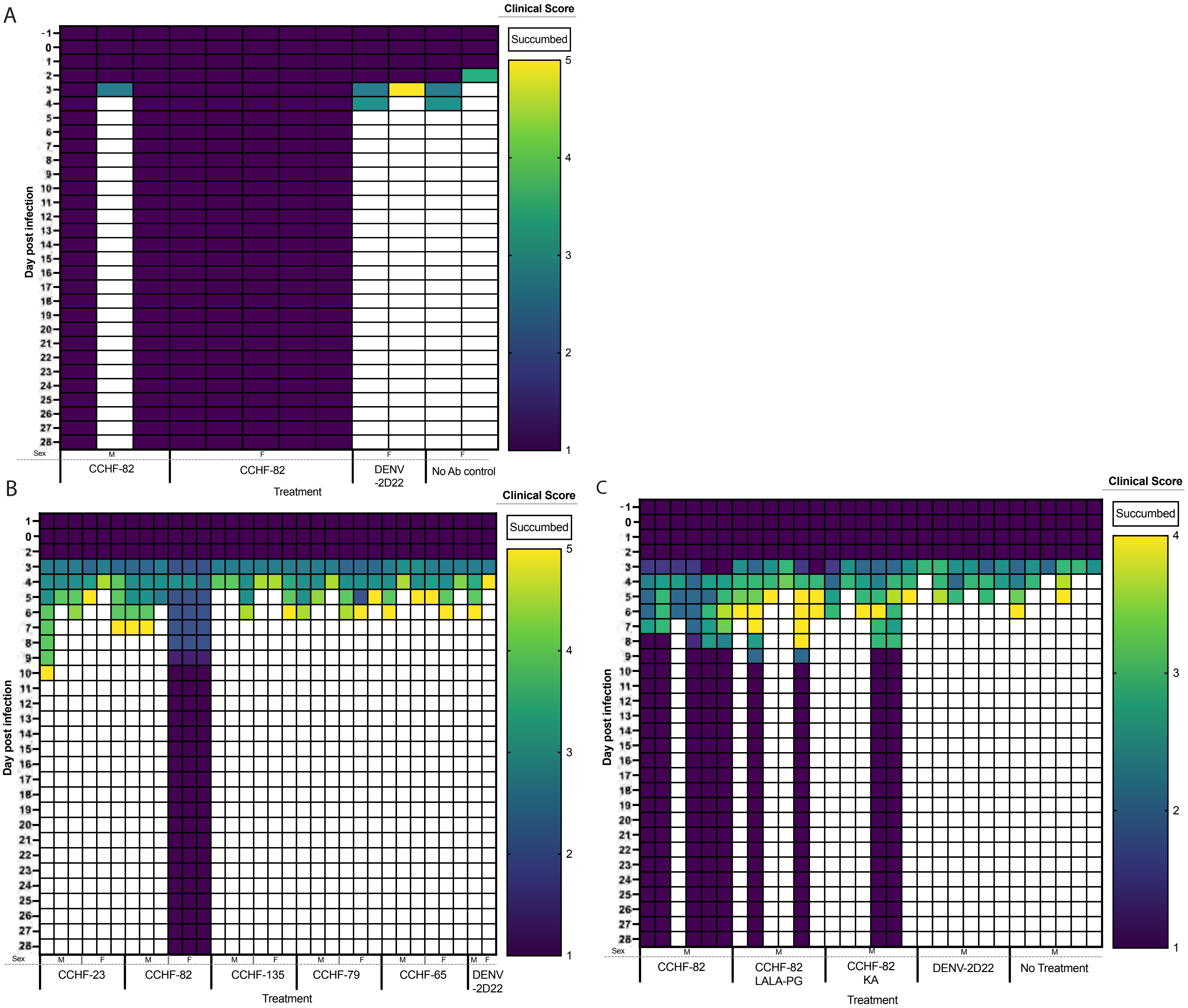

Supplemental Figure 6. Clinical scores of animals after challenge from Turkey 2004 strain of CCHFV with antibody as prophylactic or post exposure treatments.
Animals were observed on designated days during the course of their respective studies. Animals were clinically scored a value of 1 through 5. The clinical definition of each number can be found in the methods. **A**) Clinical score from the prophylactic study with CCHF-82 against the Turkish strain of CCHF. **B**) Clinical score from the post-exposure study with 5 human antibodies against the Turkish strain of CCHF. **C**) Clinical score from the mechanistic complement and Fc-effector function contribution of CCHF-82 's protective capacity study against the Turkish strain of CCHF in all male mice.

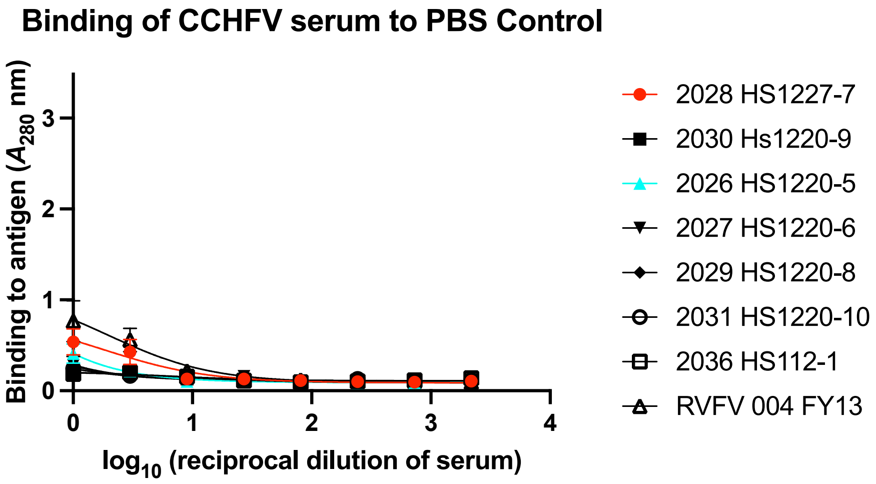

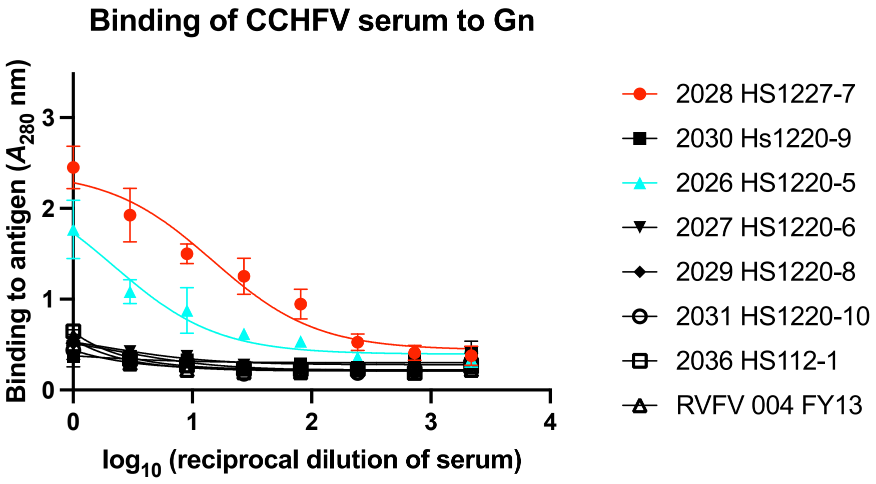

Supplemental Figure 7. Serum binding to Gn from survivors of CCHFV infection is rare but observable in select survivors.

Briefly serum was serially diluted in PBS and added to ELISA plates precoated overnight with Gn recombinant protein. Antibody was washed and secondary antibody was added. Secondary was washed off and TMB substrate was added and allowed to develop before reading at 450 nm on a plate reader. Data represents two independent experiments with three replicates each. Data were analyzed using a three-parameter nonlinear fit analysis in Prism software version 9 (GraphPad). Error bars represent mean +/- SEM.

Supplemental Tables for

Human antibody targeting Crimean-Congo hemorrhagic fever virus glycoprotein 38 protects mice against heterologous virus challenge

Nathaniel S. Chapman[1,2], Viktoriya Borisevich[3], Nurgun Kose[2], Luke Myers[2], Stephen G. Priest[2], Éric Bergeron[4,5], Elena Trigo-Esteban[6], María Paz Sánchez-Seco Fariñas[7], José Antonio Melero Fondevila[7,8,*], Thomas W. Geisbert[3,9,10], Robert W. Cross[3,9,10], James E. Crowe, Jr.[1,2,11]

This file contains:

Supplemental Tables 1-3

Supplemental Table 1. Antibody gene usage characteristics of Crimean-Congo hemorrhagic fever virus-specific human mAbs.

MAb clone, CCHF-	Heavy chain genetic features						Light chain genetic features					
	IGHV gene and allele	V-region nucleotide homology to IGHV gene, %	IGHD gene	IGHJ gene	HCDR3 amino acid (AA) sequence	HCDR3 length (AA)	Light chain type	IGKV or IGLV gene and allele	V-region nucleotide homology to IGKV or IGLV gene, %	IGKJ or IGLJ gene	LCDR3 amino acid (AA) sequence	LCDR3 length (AA)
2	*V5-51*01*	98.3	*D6-19*	*J4*	ARLPHPVTGPLDY	13	λ	*LV2-23*02*	98.3	*LJ3*	CSYAGSMTWV	10
3	*V3-21*01*	96.6	*D3-10*	*J4*	ASLRKDSGSFYNRALDY	17	λ	*LV2-14*01*	97.3	*LJ1*	YSYTSNSTYV	10
4	*3-49*04*	96.7	*D1-1*	*J4*	TRARHDTRSWVLSDH	15	κ	*KV1-5*03*	95.3	*KJ2*	QQYNSFH	7
5	*V3-43D*04*	96.9	*D3-10*	*J4*	ATGHPPLVLWSLGY	14	κ	*KV1-39*01*	96.1	*KJ5*	QQSYTTPIT	9
7	*V3-23*01*	95.6	*D2-2*	*J6*	AKRYCSGTTSHLYCYYAMDV	20	λ	*LV2-14*01*	99.3	*LJ1*	SSYTSSSTKL	10
14	*V3-21*01*	97.6	*D4-11*	*J6*	ARGGNDYSDYENYYYYMDV	19	λ	*LV3-1*01*	98.9	*LJ1*	QAWDSSTAYV	10
19	*V3-9*01*	97.3	*D3-10*	*J4*	AKDRGPFGWLTLDY	14	κ	*KV1-39*01*	96.6	*KJ1*	QQSSTTPWT	9
21	*V3-49*04*	98.7	*D3-10*	*J4*	TRATPVLLWFGSSGNFFDY	19	λ	*LV2-14*01*	99.0	*LJ1*	SSYTTSNSYV	10
	*V4-39*01*	97.0	*D3/*	*J4*	VRHGLDHKFDY	11	λ	*LV2-23*02*	96.3	*LJ2*	CSYAGGSNSVL	11

23			*OR15-3a*									
29	*V3-48*01*	98.6	*D1-1*	*J4*	ARGNGATY	8	κ	*KV1-16*02*	98.2	*KJ5*	QQYNIYPIT	9
40	*V3-9*01*	97.6	*D2-2*	*J5*	AKEDCPSTSCYFVRWGLNWLDP	22	λ	*LV1-40*01*	99.0	*LJ2*	QSFDSSLSRGV	11
50	*V1-18*01*	98.0	*D3-16*	*J4*	ARDPSMMTFGGVIVSRYFDY	20	λ	*LV1-40*01*	98.0	*LJ3*	QSYDSSVTV	9
55	*V4-39*01*	96.7	*D3/ OR15-3a*	*J5*	ARHDYWTGARYSWFDP	16	λ	*LV1-40*01*	98.7	*LJ3*	QSYDTSLSGSGV	12
59	*V1-46*01*	96.0	*D3-10*	*J6*	ARETVVQRLVGRDYYHGMDV	20	κ	*KV2-28*01*	99.3	*KJ4*	MQALQTPLT	9
61	*V1-69*01*	94.6	*D3-22*	*J2*	ARDRRYNYESSASQNNRWYFDL	22	κ	*KV4-1*01*	97.0	*KJ4*	QQYYTTPLT	9
62	*V3-30*02*	96.6	*D2-8*	*J4*	AKDLAVLLMYGFGGFDA	17	κ	*KV1-39*01*	96.1	*KJ1*	QQSYSTPVS	9
64	*V1-69*01*	98.3	*D3-3*	*J6*	ARYFFTTPHWTLPIDYGMDV	20	κ	*KV2-28*01*	98.7	*KJ2*	MQALQTPYT	9
65	*V4-4*02*	96.6	*D1-26*	*J4*	AGGTYFRRYFDY	12	κ	*KV3-15*01*	99.3	*KJ4*	QQYNNWPPLT	10
71	*V3-49*04*	96.6	*D1-1*	*J4*	LAATVWTYFDF	11	λ	*LV2-11*01*	97.0	*LJ3*	CSYAGSFTWV	10
72	*V1-18*01*	99.3	*D3-22*	*J6*	ARDYYDSSGSV	11	κ	*KV1-39*01*	97.9	*KJ1*	QQTYTTPRT	9
73	*V1-3*01*	95.6	*D1-20*	*J4*	ARVKSDTLDFNWNPRFDY	18	κ	*KV3-20*01*	96.9	*KJ5*	QQCGSSPIT	9
74	*V4-38-2*01*	98.0	*D2-2*	*J4*	ARLLPSNIY	9	λ	*LV2-11*01*	98.0	*LJ2*	CLYAGSYTFK	10

75	*V3-9*01*	95.9	*D6-13*	*J4*	AKSPLKIWQHLTPYDY	16	κ	*KV3-15*01*	99.0	*KJ4*	QQYNNWPPF	9
77	*V4-30-4*08*	97.0	*D1-26*	*J6*	ATAPAVGSYYMRWTGYHYYMDV	22	λ	*LV1-51*01*	97.3	*LJ3*	GTWDSSLNAWV	11
79	*V1-58*01*	95.9	*D2-8*	*J3*	AAGPGVWARTERPNDAFNL	19	λ	*LV1-51*01*	97.3	*LJ2*	GTWDSSLSAWI	11
82	*V4-38-2*01*	96.9	*D3-22*	*J1*	ASRHDRSGYDEYFEY	15	κ	*KV1-NL1*01*	97.2	*KJ3*	QQYYSTPLT	9
86	*3-11*01*	96.3	*D2-2*	*J5*	ARDHRYCTSTNCFAHWFDP	19	λ	*LV1-44*01*	98.0	*LJ3*	AAWDDSLNGPV	11
88	*V1-46*03*	92.9	*D1-26*	*J4*	ARWGLISESSPKYFDS	16	λ	*LV1040*01*	98.0	*LJ2*	*QSYDSSVSGFYVL*	13
95	*V4-4*02*	95.3	*D6-13*	*J5*	ARAGLYSTNWSPFDP	15	κ	*KV3-20*01*	97.6	*KJ1*	QQYGGSPWT	9
104	*V3-11*01*	95.9	*D6-19*	*J4*	ARSLRGIAVPSY	12	κ	*KV3-15*01*	97.9	*KJ1*	QQYNNWPPWT	10
105	*V4-39*01*	95.7	*D2-8*	*J5*	ASQKMVYPIKRNNWFDP	17	λ	*LV1-44*01*	98.0	*LJ3*	AAWDDGLNGWV	11
106	*V5-51*01*	99.3	*D3-3*	*J6*	ARHESEAFSIFGVVRYYYYYMDV	23	λ	*LV2-11*01*	99.3	*LJ2*	CSYAGTVV	8
108	*V4-4*02*	97.3	*D6-19*	*J6*	ARVGLGWHDGNGMDV	15	κ	*KV3-11*01*	95.8	*KJ2*	QQRSNWPPGYT	11
114	*4-31*03*	95.3	*D5-24*	*J4*	ARFRLGDAPTRDGYNLHYFDY	21	λ	*LV2-23*02*	98.0	*LJ2*	CSYGGFSTHVV	11
115	*V3-49*04*	94.4	*D3-16*	*J4*	TRAHYDYVWGNYWSFAY	17	λ	*LV1-51*01*	98.0	*LJ1*	GTWDSSLSVFV	11
116	*V3-33*01*	97.6	*D5-24*	*J3*	ARDPGGRRDGYILRPDAFDI	20	κ	*KV1-5*03*	98.9	*KJ1*	QQYNTYTWT	9

117	*V1-18*01*	99.3	*D5-12*	*J4*	ARSGGYAIF	9	κ	*KV3-20*01*	98.6	*KJ2*	QQYGSTPPYT	10
131	*V5-51*01*	98.0	*D5-24*	*J6*	ARHSETKDGYNWAQGNFYSYYYMDV	25	κ	*KV1-39*01*	97.9	*KJ4*	QQSYSISPLS	10
132	*V4-59*01*	93.7	*D5-12*	*J4*	ARDSRRNRYSGYYFDF	16	κ	*KV3-15*01*	97.2	*KJ1*	HQYNNWPQT	9
135	*V1-24*01*	93.2	*D6-19*	*J4*	ATDPGAVAGFLGF	13	κ	*KV4-1*01*	97.3	*KJ3*	QQYYGTVT	8
137	*3-49*04*	97.3	*D3-9*	*J4*	LAGTDWSYFDY	11	λ	*LV2-11*01*	98.6	*LJ3*	CSYAGSYTWV	10
144	*V4-59*01*	96.6	*D3-10*	*J6*	ARGAYYGSGSFHYYYYMDV	19	κ	*KV1-5*03*	96.5	*KJ2*	QQYNGYSYT	9
190	*V3-7*01*	99.0	*D2-2*	*J4*	ASYCSSTSCHIDPIDY	16	κ	*KV1-39*01*	97.2	*KJ1*	QQGYSTPRT	9
192	*V3-23*01*	94.9	*D2-8*	*J4*	AKPDCTSYRCYMLSHD	16	κ	*KV1D-16*01*	99.3	*KJ4*	QQYSSYPLT	9
193	*V1-46*03*	95.3	*D1-20*	*J4*	ARGSTGITGDPHYFDF	16	κ	*KV1-16*02*	97.2	*KJ2*	QQYFRYPPT	9
194	*V4-4*02*	96.5	*D6-13*	*J3*	RISNWFGPYDAFDI	14	κ	*KV3-20*01*	97.9	*KJ3*	QQHGSSRT	8
196	*V1-46*01*	97.3	*D2-2*	*J2*	ARGGSITTPQGWYFDL	16	λ	*LV1-40*01*	98.0	*LJ2*	QSYDNSLSGWDVV	13
202	*V3-9*01*	96.0	*D2-2*	*J5*	VKDASSRIYQLSRWFDP	17	κ	*KV3-15*01*	96.5	*KJ2*	QQYNNWPRT	9
206	*V4-39*01*	96.9	*D1-26*	*J4*	VEGGSYPYYFDY	12	κ	*KV1-39*01*	98.2	*KJ3*	QQSYSTLFT	9
207	*V3-30*02*	95.2	*D5-18*	*J4*	AHLEAYSYVFSL	12	κ	*KV2-30*01*	98.3	*KJ2*	MQGTHWPPYT	10

208	*V4-38-2*01*	96.9	*D1-26*	*J4*	ARHLGEWELLPIDY	14	κ	*KV3-20*01*	98.3	*KJ4*	QQYGSSPPT	9
211	*V4-39*01*	97.6	*D6-13*	*J6*	ASPPSGSSSWFRNHYYMDV	19	κ	*KV3-20*01*	99.0	*KJ1*	QEHGSSPSLT	10
213	*V4-38-2*01*	99.7	*D3-22*	*J3*	ARKSVFGYYDTSGYYSAFDI	20	κ	*KV3-20*01*	99.6	*KJ1*	QQYGSSPVT	9
225	*V4-39*01*	95.3	*D3-3*	*J4*	ARHVITISGVIRGFDY	16	λ	*LV2-8*01*	97.0	*LJ1*	CSYAGTLSWAV	11
227	*V4-61*01*	95.6	*D2-15*	*J4*	ARGGIYCDGPGCYWLAPDY	19	λ	*LV1-47*01*	96.6	*LJ2*	ASWDDSLSGHVV	12
229	*V4-34*02*	93.5	*D5-18*	*J4*	ARGSGHSWLRGSHFDH	16	κ	*KV3-15*01*	97.6	*KJ1*	HQYNNWPPWT	10
236	*V1-3*01*	97.3	*D3-22*	*J3*	ARGARFSMRVVIVTNTFDI	19	κ	*KV1-33*01*	99.0	*KJ3*	QHYDNLLSFT	10
239	*V3-30*18*	95.6	*D4-23*	*J6*	AKDRYYGANWGPGHYGMDV	19	κ	*KV3-20*01*	97.2	*KJ3*	QQYGSSPFT	9
240	*V1-24*01*	97.6	*D5-18*	*J4*	ATPTSSGYSYGYFFNY	16	κ	*KV3-15*01*	99.3	*KJ1*	QQYNNWPPWT	10
243	*V2-5*02*	95.6	*D2-2*	*J4*	SRWTLDLLVVPAPTYFDY	18	κ	*KV1-39*01*	95.1	*KJ1*	QQSSTSPWT	9
245	*V3-30*02*	97.3	*D1-26*	*J3*	ATPSGSSGAFDF	12	κ	*KV3-15*01*	97.9	*KJ1*	QQYNNWPPERT	11
252	*V1-69*01*	94.3	*D5/OR1<br>5-5a*	*J4*	ARDGRAVSFRSYFES	15	λ	*LV1-40*01*	97.6	*LJ3*	QSYDSSLTGKV	11
259	*V4-4*02*	97.6	*D3-10*	*J6*	ARKNLPVLLWFGAGDYYYMDV	21	κ	*KV2-28*01*	99.7	*KJ5*	MQALQTPPIT	10
263	*V1-46*01*	97.3	*D1-1*	*J4*	ARDPTKNERRGGPYYFDF	18	λ	*LV3-25*02*	97.9	*LJ2*	QSADSSGTYHVI	12

268	*V3-30*02*	96.2	*D7-27*	*J4*	AKNRPLGITDY	11	κ	*KV3-15*01*	98.6	*KJ5*	QQYSNWPPIT	10
275	*V3-23*01*	99.0	*D1-1*	*J4*	AKGPGGSGFDY	11	κ	*KV3-15*01*	99.7	*KJ4*	QQYNNWPPFT	10

Supplemental Table 2. Binding of human mAbs to cell-surface displayed glycoproteins for diverse CCHFV strains

MAb (CCHFV-)	Half-maximal effective concentration (EC_{50}) value [ng/mL] for binding of mAb to indicated CCHFV strain ***										
	IbAr10200	TURK	Sudan	Oman	VC04	SPU18	NIV	Hoti	ARD	AFG09	BAGH	LASV II
2	56	155	278	151	195	161	109	224	122	123	107	>
23	82	73	77	68	90	94	46	88	70	62	45	>
59	2,203	305	543	262	458	430	281	433	307	509	299	>
61	146	19,000	412	319	535	240	171	1,073	940	325	143	>
65	447	394	358	538	263	625	290	529	334	339	224	>
79	53	130	137	105	108	166	109	135	140	116	93	>
82	246	306	496	510	643	963	915	789	303	213	5	>
115	537	1,132	1,541	1,732	702	1,563	849	1,067	975	1,056	701	>
117	146	483	556	334	543	459	309	553	443	463	317	>
137	171	228	546	175	555	390	177	437	229	497	268	>

*** Binding assay was performed three times with technical duplicates in each assay. Results were similar between biological replicates; data shown are the mean values of technical replicates as an average from all three assays.

> Indicates binding was not detected even at the highest concentration tested of 30 µg/mL.

Supplemental Table 3. Neutralization of authentic CCHFV strain IbAr10200 or CCHFV transcription- and entry-competent VLPs (tecVLPs) by human mAbs

MAb (CCHF-)	Half-maximal inhibitory concentration (IC_{50}) value for neutralization by human mAbs ***								
	Using authentic virus (biosafety level 4)	Using tecVLPs for indicated CCHFV strain (biosafety level 2) [ng/mL]							
	IbAr10200 [µg/mL]	IbAr10200	Sudan	ARD	VC04	SPU18	NIV	Oman	Turk
2	>	<40	337	<40	126	27	1,919	188	407
23	0.3	<40	<40	<40	<40	<40	<40	27	137
59	3,051	331	305	656	384	735	113	324	895
61	>	145	454	2,147	242	24	401	101	5,336
65	>	105	592	674	139	52	271	188	798
79	335	106	237	1.8	34	<40	<40	23	224
82	>	>	>	>	>	>	>	>	>
115	>	794	349	1,142	1,101	1,645	754	2,583	1,240
117	8,075	100	339	13.6	49	99	167	<40	288
137	2,200	139	136	95	290	157	28	52	246

*** Neutralization assay was performed three times with technical duplicates in each assay. Results were similar between biological replicates; data shown are the mean values of technical replicates as an average from all three assays. The authentic virus neutralization assay values represent a mean value of technical duplicates from an assay that was performed once with duplicate values.

> Indicates neutralization was not detected even at the highest concentration tested of 30 µg/mL.
